# Characterization and Interaction Analysis of the Secondary Cell Wall Synthesis-Related Transcription Factor *PmMYB7* in *Pinus massoniana* Lamb.

**DOI:** 10.3390/ijms23042079

**Published:** 2022-02-14

**Authors:** Peizhen Chen, Rong Li, Lingzhi Zhu, Qingqing Hao, Sheng Yao, Jiahe Liu, Kongshu Ji

**Affiliations:** Key Laboratory of Forest Genetics & Biotechnology of Ministry of Education, Co-Innovation Center for the Sustainable Forestry in Southern China, Nanjing Forestry University, Nanjing 210037, China; pei_jane@126.com (P.C.); hblxylr@126.com (R.L.); lingzhi_zhu@126.com (L.Z.); hqq@njfu.edu.cn (Q.H.); yaosheng0827@126.com (S.Y.); xiaohecljjj@njfu.edu.cn (J.L.)

**Keywords:** CCoAOMT2, lignin, MYB7, protein–protein interaction, *Pinus massoniana*, secondary cell wall, transcriptional regulation

## Abstract

In vascular plants, the importance of R2R3-myeloblastosis (R2R3-MYB) transcription factors (TFs) in the formation of secondary cell walls (SCWs) has long been a controversial topic due to the lack of empirical evidence of an association between TFs and downstream target genes. Here, we found that the transcription factor *PmMYB7*, which belongs to the R2R3-MYB subfamily, is involved in lignin biosynthesis in *Pinus massoniana*. *PmMYB7* was highly expressed in lignified tissues and upon abiotic stress. As a bait carrier, the PmMYB7 protein had no toxicity or autoactivation in the nucleus. Forty-seven proteins were screened from the *P. massoniana* yeast library. These proteins were predicted to be mainly involved in resistance, abiotic stress, cell wall biosynthesis, and cell development. We found that the PmMYB7 protein interacted with caffeoyl CoA 3-O-methyltransferase-2 (PmCCoAOMT2)—which is involved in lignin biosynthesis—but not with beta-1, 2-xylosyltransferase (PmXYXT1) yeast two-hybrid (Y2H) studies. Our in vivo coimmunoprecipitation (Co-IP) assay further showed that the PmMYB7 and PmCCoAOMT2 proteins could interact. Therefore, we concluded that *PmMYB7* is an upstream TF that can interact with PmCCoAOMT2 in plant cells. These findings lay a foundation for further research on the function of *PmMYB7*, lignin biosynthesis and molecular breeding in *P. massoniana.*

## 1. Introduction

**Figure 1:**
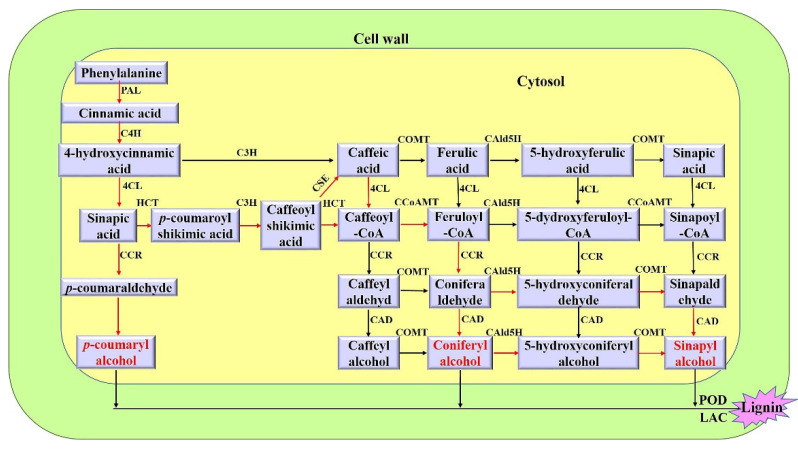
The general lignin biosynthesis pathway [6,7]. The red arrows indicate the main metabolic flux. PAL, phenylalanine ammonia-lyase; C4H, cinnamate 4-hydroxylase; C3H, p-coumaroyl-CoA 3-hydroxylase; 4CL, p-coumarate CoA ligase; HCT, hydroxycinnamoyltransferase; CSE, caffeoyl shikimate esterase; CCoAOMT, caffeoyl-CoA O-methyltransferase; CCR, cinnamoyl-CoA reductase; CAld5H, coniferaldehyde 5-hydroxylase; COMT, caffeic acid 3-O-methyltransferase; CAD, cinnamyl alcohol dehydrogenase; POD, peroxidase; LAC, laccase. p-coumaryl alcohol (H monolignol), coniferyl alcohol (G monolignol) and sinapyl alcohol (S monolignol) are marked in red.

The complex structure of the plant cell wall differs among different species and tissue types. Wood formation in perennial woody species is a dynamic and continuous process requiring a large number of fixed carbon resources to enter the lignin-building modules, which are involved in cambium cell proliferation, xylem cell differentiation, secondary cell wall (SCW) biosynthesis and programmed cell death (PCD) [1,2]. SCWs, which are thicker than the primary cell wall (PCW), are deposited between the PCW and the plasma membrane during the later stages of the cessation of cell growth and division [3]. This SCW mainly includes lignin, cellulose and hemicellulose, which play important roles in the development, mechanical support and waterproofness of vessel walls, as well as in the stress responses of plants [3,4]. Lignin is oxidatively polymerized primarily from three types of monolignols (p-coumaryl alcohol, coniferyl alcohol and sinapyl alcohol) that are synthesized in the cytosol from phenylalanine through successive deamination, reduction, hydroxylation and methylation processes (Figure 1) [5,6,7].

Myeloblastosis (MYB) transcription factors (TFs), characterized by highly conserved MYB DNA-binding domains, can be classified as 1R-MYB, R2R3-MYB, 3R-MYB and 4R-MYB proteins [8]. As one of the largest families of TFs, R2R3-MYB are involved in the transcriptional control of development, secondary metabolism and responses to biotic and abiotic stresses, as has been well demonstrated in *Arabidopsis thaliana*, *Eucalyptus grandis*, *Paulownia tomentosa, Gossypium hirsutum* and other plants [9,10,11,12,13]. In recent years, it has been reported that some MYB TFs are involved in the formation of the xylem or phloem and that they may control the accumulation of biomass by regulating the expression of TFs during the secondary growth of plants [14]. For example, *PttMYB21a* negatively regulates caffeoyl CoA 3-O-methyltransferase (*CCoAOMT)* expression and the lignin content in the secondary walls of *Populus tremula × tremuloides* [15]. Constitutive expression of *GhMYB7* in *A. thaliana* activates the expression of a series of SCW biosynthesis-related genes, leading to the ectopic deposition of cellulose and lignin [10]. Moreover, *GhMYB7* binds to the promoter regions of staphylococcal nuclease and tudor domain containing 1 (*AtSND1*) and cellulose synthase 4 (*AtCesA4*) and participates in the regulation of cotton fiber SCW biosynthesis [10]. *AtMYB7* can shut down genes required for lignin biosynthesis and inhibit the pathway in *A. thaliana* [16]. In *Eucalyptus*, *EgMYB1* negatively regulates SCW formation in *A. thaliana* and poplar, whereas *EgMYB2* positively regulates SCW formation in tobacco [17].

However, only a few functional studies have examined the role of R2R3-MYB TFs in gymnosperms. For example, loblolly pine *PtMYB1* can bind to cis-acting AC elements to activate the phenylalanine ammonia-lyase 2 (*PAL2*) promoter and participate in the regulation of lignin biosynthesis [18]. The spruce R2R3-MYB TFs *PgMYB2*, *PgMYB4* and *PgMYB8* are preferentially expressed during the differentiation of the xylem of the stem and root. During the induction of compression wood, the transcription level of some MYB genes and the expression of cell wall-related genes are upregulated during the differentiation of the secondary xylem from saplings [19]. Loblolly pines *PtMYB1* and *PtMYB8* may rely on the information conserved about the transcription regulatory network involved in SCW deposition in conifers. Overexpression of *PtMYB1* and *PtMYB8* leads to the simultaneous upregulation of many genes involved in lignin monomer synthesis in *Pinus taeda* [20]. Lignin deposition increases in transgenic tobacco plants that overexpress *PtMYB4* and extends to cell types that are not normally lignified. Therefore, *PtMYB4* is sufficient to induce lignification and may play a role in the wood formation process of pine [21]. In addition, *PpNAC1* (NAM, ATAF and CUC, the name having been derived from the first three reported members of the family, NO APICAL MERISTEM/NAM, *A. thaliana* Activation Factor1 and 2/ATAF1/2 and CUP-SHAPED COTYLEDON 2/CUC2) activates its own expression and the *PpMYB4* promoter, while *PpMYB4* can activate the expression of *PpMYB8* and lignin biosynthesis in *Pinus pinaster* [22]. Analyzing the molecular switch that controls the formation of wood is of vital importance for understanding tree biology and laying a solid foundation for biotechnological applications in conifers.

*Pinus massoniana* is an important industrial and ecological evergreen tree species in China. It is distributed in the middle and lower reaches of the Yangtze River and in southern China. *P. massoniana* is an excellent pulp production plant with high cellulose and lignin levels [23]. It has always been considered the main wood resource for the fiber (for pulp, paper products and boards) and sawn timber (for building houses and furniture) industries in China [24]. However, lignin is a major barrier to applications related to pulp, paper and chemical feedstock production. The content, composition and structure of lignin have effects on the process efficiency of woody biomass [25,26]. Hence, reducing the lignin content is one of the main methods for improving the utilization of *P. massoniana* pulp. The synthesis of lignin is part of the phenylpropanoid metabolic pathway. In addition to the enzymes involved in the lignin biosynthetic pathway, upstream TFs also exert a great effect on the biosynthesis of lignin [27]. However, little is known about the key TFs involved in the biosynthetic pathways of SCW in *P. massoniana*. In this study, to expand our understanding of SCW formation, we carried out a functional analysis of MYB genes. Based on this conserved regulatory pattern between angiosperms and gymnosperms, we identified *PmMYB7*. Our expression analysis demonstrated that *PmMYB7* was highly expressed in lignified tissues and under abiotic stress conditions. By constructing a nuclear system yeast library, a set of genes involved in resistance to pathogens, abiotic stress, cell wall biosynthesis and cell development were predicted and screened out. Further yeast two-hybrid and coimmunoprecipitation (Co-IP) assays showed that *PmMYB7* is an upstream TF that interacts with the PmCCoAOMT2 protein and participates in regulating lignin biosynthesis.

## 2. Results

### 2.1. Full-Length Cloning and Bioinformatics Analysis of the PmMYB7 Gene Coding Sequence

**Figure 2:**
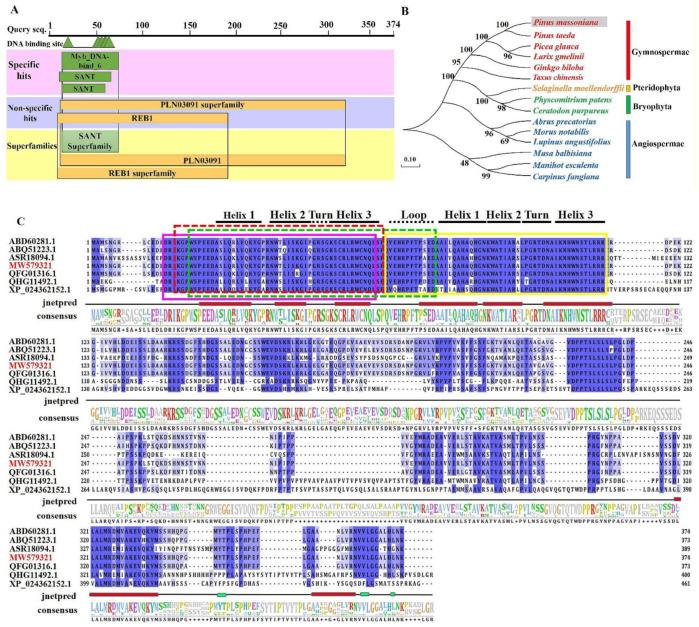
Structural features and phylogenetic analysis of the PmMYB7 protein. (**A**) Structure of the *PmMYB7* protein. (**B**) Phylogenetic analysis of *PmMYB7* with R2R3-MYB TFs. Red represents Gymnospermae, yellow Pteridophyta, green Bryophyta, and blue Angiospermae. *P. massoniana* in gray shadow represents PmMYB7. The GenBank accession numbers are as follows: *P. taeda* (ABD60281.1), *Picea glauca* (ABQ51223.1), *Larix gmelinii* (QFG01316.1), *Ginkgo biloba* (ASR18094.1), *Taxus chinensis* (QHG11492.1), *Selaginella moellendorffii* (XP_024536333.1), *Physcomitrium patens* (XP_024362152.1), *Ceratodon purpureus* (KAG0581796.1), *Abrus precatorius* (XP_027345753.1), *Morus notabilis* (XP_010095320.2), *Lupinus angustifolius* (XP_019444671.1), *Musa balbisiana* (THU58269.1), *Manihot esculenta* (XP_021601648.1) and *Carpinus fangiana* (KAE8100124.1). (**C**) Amino acid sequence alignment of *PmMYB7* from *P. massoniana* and other plants. The R2 domain is indicated by a pink box, and the R3 domain by a yellow box. “jnetpred” in the picture represents the predicted secondary structure of the MYB7 protein: α-helices are represented by red tubes, and β-sheets are represented by green tubes. The MYB-binding domain is indicated by a green dotted line box. The SANT domain is indicated by a red dotted line box. MW579321 in red represents PmMYB7.

Based on the GenBank database, *P. massoniana* transcriptomic data (PRJNA655997) and bioinformatics analysis [28], we chose a sequence that included three domains, namely, the MYB-binding domain, the SANT (SWI3, ADA2, N-CoR and TFIIIB B) domain and the helix-helix-turn-helix domain that are necessary for the functional MYBs protein [8], and further cloned these sequences. After comparison with the *PtMYB7* coding sequence of *P. taeda*, which is closely related to *P. massoniana,* one of the coding sequences was named *PmMYB7*. The full-length cDNA of the *PmMYB7* gene is 1665 bp in length. The putative open reading frame (ORF) of *PmMYB7* is 1125 bp in length and encodes 374 amino acids (Supplementary S1). The estimated molecular of the protein and its theoretical isoelectric point (pI) are 40.78 kDa and 7.72, respectively. Using the NCBI Conserved Domain database, we found that the conserved domains of the protein include the MYB-binding domain at 19~76 aa and the SANT domain at 16~64 aa, and *PmMYB7* belongs to the SANT protein superfamily (Figure 2A).

To further explore the phylogenetic relationships of the PmMYB7 protein, we performed a BLAST search against the NCBI protein database. We compared 15 protein sequences with high similarity to the PmMYB7 protein, which were distributed into four different groups, namely, Gymnospermae, Pteridophyta, Bryophyta and Angiospermae, and the sequence of PmMYB7 clustered together with that of *P. taeda* (Figure 2B). By using the DNAMAN software, we found that the PmMYB7 protein contains complete R2 and R3 areas. The R2 and R3 repeat areas have a primary structure, (-W-(X19)-W-(X19)-W-) and (-F/I/L/M-(X18)-W-(X18)-W-), and a secondary structure, (helix-helix-turn-helix) (Figure 2C). Therefore, *PmMYB7* belongs to the R2R3 subfamily of MYB TFs.

### 2.2. Expression Profiling of the PmMYB7 Gene

**Figure 3:**
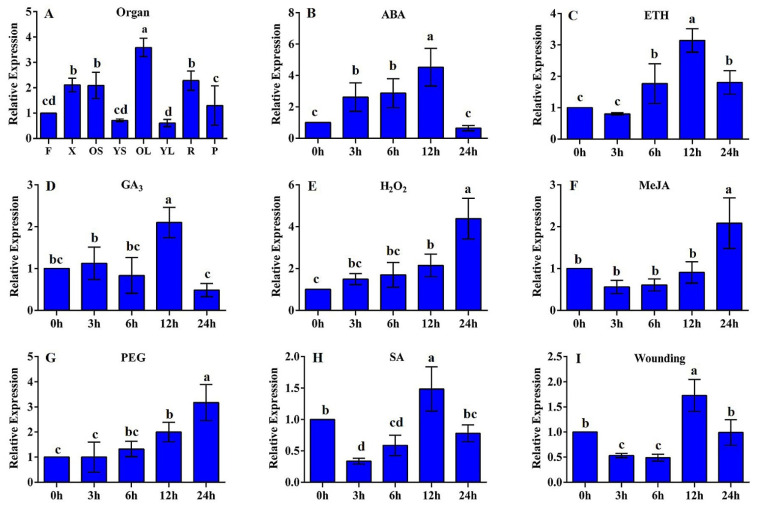
Expression patterns of the *PmMYB7* gene as determined by RT–qPCR. (**A**) Expression levels in different organs/tissues. F, flowers; X, xylem; OS, old stems; YS, young stems; OL, old leaves; YL, young leaves; R, roots; P, phloem. (**B**–**I**) *PmMYB7* expression levels in *P. massonniana* under different stress conditions. All the above treatments were added at 0 h, and no treatment was used for the control. Figure (**B**) shows the relative expression levels under 400 μM ABA conditions; figure (**C**) shows the relative expression under 50 μM ETH treatment conditions; figure (**D**) shows the relative expression under 2 mM GA_3_ treatment conditions; figure (**E**) shows the relative expression under 10 mM H_2_O_2_ treatment conditions; figure (**F**) shows the relative expression under 10 mM MeJA treatment conditions; figure (**G**) shows the relative expression under 15% PEG 6000 treatment conditions; figure (**H**) shows the relative expression under 1 mM SA treatment conditions; figure (**I**) shows the relative expression under wounding treatment conditions. The error bars represent the standard deviation of three biological replicates. Means with different letters are significantly different at *p* < 0.05.

The expression patterns showed that the *PmMYB7* gene was expressed in all the examined organs and tissues. However, the expression level in old leaves was the highest, followed by that in xylem, old stems and roots. The expression levels were lowest in the young stems and young leaves (Figure 3A). After treatment with abscisic acid (ABA), ethephon (ETH) and gibberellin (GA), the expression level of the *PmMYB7* gene was increased and then decreased, peaking at 12 h; compared with the control group, the expression increased by 4.52-fold, 3.14-fold and 2.09-fold (Figure 3B–D), respectively. However, in response to treatment with H_2_O_2_, methyl jasmonate (MeJA) and polyethylene glycol (PEG 6000), the expression level of *PmMYB7* peaked at 24 h and was 4.38 times, 2.08 times and 3.17 times higher, respectively, than after the control treatment (Figure 3E–G). After treatment with salicylic acid (SA) and wounding, the expression level of the *PmMYB7* gene decreased, increased and then decreased again, peaking at 12 h; the peak expression level under SA and wounding conditions was 1.49 times and 1.73 times higher, respectively, than that under control conditions (Figure 3H,I).

### 2.3. Subcellular Localization of the PmMYB7 Protein

**Figure 4:**
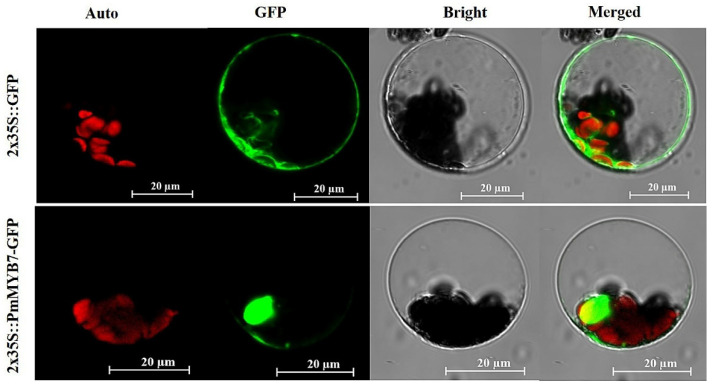
Subcellular localization analysis of 2x35S::PmMYB7-GFP in *A. thaliana*. The 2x35S::GFP was used as a control protein. Auto, chlorophyll autofluorescence; GFP, green fluorescent protein; Bright; Merged, GFP + Bright + Auto; Scale bar, 20 μm.

The Cell-PLoc prediction results indicate that the PmMYB7 protein is a nuclear protein. The subcellular localization results show that the 2x35S::PmMYB7-GFP (green fluorescent protein) protein was transiently expressed in the leaf protoplasts of *A. thaliana*. Compared with the control 2x35S::GFP signal, the 2x35S::PmMYB7-GFP signal was mainly emitted from the nucleus (Figure 4).

### 2.4. Construction of a P. massoniana Yeast cDNA Library

**Figure 5:**
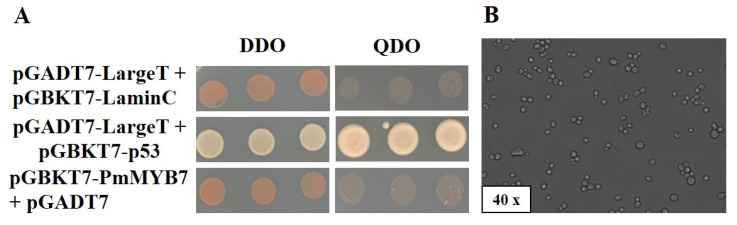
Autoactivation and toxicity analysis of pGBKT7-PmMYB7 and library screening. (**A**) AuTable 7. p53 + pGADT7-largeT was cotransformed as a positive control, and pGBKT7-laminC + pGADT7-largeT was cotransformed as a negative control. (**B**) The diploid yeast cells were grown to the typical clover-leaf-shape stage.

The lengths of the inserted fragments ranged from 650 bp to 3000 bp, with a recombination efficiency of 98% (Figure 5A). The library capacity was determined by using a 10,000-fold dilution of the transformed bacteria. The total capacity was 514/10 × 10,000 × 10^3^ CFU/mL × 5 mL = 2.57 × 10^9^ CFU (Supplementary S2). Seven of the twenty-two positive colonies were successfully sequenced and blasted against the NCBI database. The results indicated that all of the sequences were homologous to the corresponding proteins in *Populus alba* (XM_035039958.1, XM_035035016.1, XM_035051360.1, XM_035038801.1, XM_035076890.1, XM_035042975.1 and XM_035072992.1) (Supplementary S3). The plasmid library was transformed into *S. cerevisiae* AH109 to obtain a yeast library, and 1000-fold diluted yeast cells were grown on yeast peptone dextrose adenine medium (YPDA) plates. The total yeast library capacity was 2600/10 × 1000 ×10^3^ CFU/mL × 5 mL = 1.3 × 10^9^ CFU (Supplementary S2).

### 2.5. Autoactivation and Toxicity Analysis of Bait Vectors and Library Screening

We used yeast two-hybrid technology to screen interacting proteins, identify protein interactions and explore the mechanisms underlying protein–protein interactions. The positive control and negative control strains grew on SD/-Trp/-Leu (DDO) plates, and the positive control, but not the negative control, grew on SD/-Trp/-Leu/-His/-Ade (QDO) plates. The experimental group pGBKT7-PmMYB7 + pGADT7 grew in a manner that was consistent with the negative control, indicating that PmMYB7 could not activate the *HIS3* and *ADE2* reporter genes (Figure 5A). Therefore, there was no autoactivation of the PmMYB7 protein. The recombinant plasmid pGBKT7-PmMYB7 could be grown on DDO plates with the prey vector pGADT7, suggesting that it could be successfully transformed into host cells without causing toxicity. When the bait vector pGBKT7-PmMYB7 and the AH109 yeast library grew into a typical clover-leaf shape, the yeast zygotes were cultured on SD/-Trp/-Leu/-His (TDO) and SD/-Trp/-Leu/-His/X-α-Gal (TDO/X) plates for selection (Figure 5B). Forty-seven out of the sixty-three screened positive colonies on the TDO plate were successfully sequenced and blasted against the National Center for Biotechnology Information (NCBI) database. The functions of the 47 proteins were predicted using the NCBI and STRING databases. The results showed that these proteins are encoded by different genes and participate in a variety of biological processes (Table 1) mainly including proteins related to resistance, abiotic stresses, protein biosynthesis and cell development.

### 2.6. Protein–Protein Interaction Networks of A. thaliana MYBR1 (MYB44) Transcription Factor

**Figure 6:**
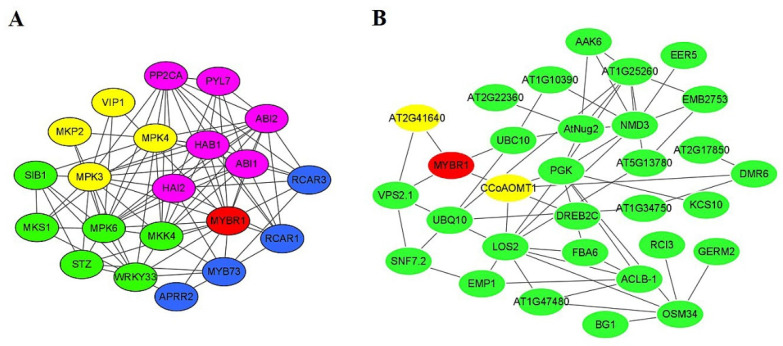
Protein–protein interaction networks of *A. thaliana* MYBR1 transcription factor predicted by STRING (**A**) and Cytoscape (**B**). In the STRING-based network, proteins involved in defense responses and enzyme regulation are shown in green; proteins involved in ABA receptor pathways are shown in pink; proteins involved in kinase signaling transduction and activation are shown in yellow; proteins involved in stress responses are shown in blue. In the Cytoscape-based network, CCoAOMT1 protein and AT2G41640 protein in yellow represent homologous proteins of CCoAOMT2 and XYXT1, respectively.

According to the classification of R2R3-MYB of *A. thaliana*, Bedon et al. have evolutionarily classified PtMYB7 and PgMYB7 and found them clustered together with AtMYB44 (MYBR1) and belonging to the 22nd subgroup [18]. In this study, we obtained that the similarity of PtMYB7 and PmMYB7 reached 99%. So, MYBR1 represents the closest homolog of the *P. massoniana* protein in *A. thaliana*. We built MYBR1 protein signaling networks for MYBR1 with String and Cytoscape. Yet, this similarity does not necessarily mean that PmMYB7 will have absolutely the same interaction partners as MYBR1. As shown in Figure 6A, STRING online software predicted that the MYBR1 protein interacted with proteins such as WRKY33, MKK4 and STZ, which are involved in defense responses and enzyme regulation (marked in green). The MYBR1 protein was also observed to interact with the ABA receptor protein PYL7 (marked in pink) and stress response proteins, such as MYB73, APRR2, RCAR1 and RCAR3 (marked in blue). The kinase signal transduction and activation proteins MPK3 and VIP1 (marked in yellow) interacted with the MYBR1 protein. This kind of predicted interaction is a co-expression network. In the screening results (Table 1), the homologous proteins of 30 proteins interacted with the MYBR1 protein in *A. thaliana*. Among them, the MYBR1 protein interacted with the CCoAOMT1 protein and the AT2G41640 protein, which are homologous proteins of CCoAOMT2 and XYXT1, respectively (Figure 6B). The interaction with other pathway proteins (MYBR1 with CCoAOMT1) will be interesting associations for studying the lignin biosynthesis pathway. The association of PmMYB7 with downstream proteins will probably provide more insights into other biosynthetic pathways of *P. massoniana*.

### 2.7. Validation of Interacting Proteins

**Figure 7:**
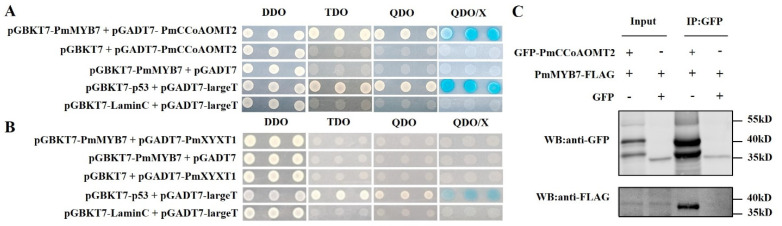
Validation of the interaction proteins. (**A**) Point-to-point validation of the interaction of pGBKT7-PmMYB7 + pGADT7-PmCCoAOMT2. pGBKT7-p53 + pGADT7-largeT was cotransformed as a positive control, and pGBKT7-laminC + pGADT7-largeT was cotransformed as a negative control. (**B**) Point-to-point validation of the interaction of pGBKT7-PmMYB7 + pGADT7-PmXYXT1. (**C**) Detection of PmMYB7 protein and PmCCoAOMT2 protein interactions in *N. benthamiana* leaves by Co-IP.

We successfully constructed the pGADT7-PmCCoAOMT2 and pGADT7-PmXYXT1 vectors and performed point-to-point yeast transformation verification with pGBKT7-PmMYB7. The positive control strains grew normally on DDO, TDO, QDO and SD/-Trp/-Leu/-His/-Ade/X-α-Gal (QDO/X) plates and developed color on the QDO/X plate. The negative control strains grew on the DDO plate but not on the TDO, QDO or QDO/X plates. In the experimental groups, the growth of pGBKT7-PmMYB7 + pGADT7-PmCCoAOMT2 was consistent with that of the positive control (Figure 7A), while the growth of pGBKT7-PmMYB7 + pGADT7-PmXYXT1 was the same as that of the negative control (Figure 7B). Accordingly, the pGBKT7-PmMYB7 protein interacted with the pGADT7-PmCCoAOMT2 protein, and the pGBKT7-PmMYB7 and pGADT7-PmXYXT1 proteins did not interact.

To further explore the interaction between PmMYB7 and PmCCoAOMT2 in plant cells, we transiently expressed fusion proteins in *Nicotiana benthamiana* leaves and identified them by coimmunoprecipitation (Co-IP). To avoid the interference caused by the MYB7 or CCoAOMT2 homologous proteins expressed in tobacco and improve the specificity of detection, both of these proteins were fused with protein tags to obtain PmMYB7-FLAG and GFP-PmCCoAOMT2 fusion proteins, respectively. The two fusion proteins described above served as the experimental group, and the mixed injection of PmMYB7-FLAG and GFP was the control group to exclude the possibility of interaction between PmMYB7 and GFP tags. As shown in Figure 7C, bands of PmMYB7-FLAG, GFP-PmCCoAOMT and GFP could be detected in the input sample, indicating that the three kinds of proteins were present in the input sample. In the GFP IP samples, obvious PmMYB7-FLAG fusion protein bands were detected by anti-FLAG in the experimental group, while no PmMYB7-FLAG bands were detected in the control group, indicating that PmMYB7 could interact with PmCCoAOMT2 in plant cells and that the interaction was specific rather than caused by the GFP fusion tag. The subcellular localization prediction of PSORT showed that the PmCCoAOMT protein was mainly located in the cytoplasm.

## 3. Discussion

### 3.1. R2R3-MYB and Characterization of PmMYB7 in P. massoniana

R2R3-MYB proteins with different functions and expression levels have been identified in different species. Fan et al. [29] identified 59 *PmMYBs* among the known sequence of *P. massoniana*, including 39 typical R2R3-MYB TFs. Compared with the numbers in *A. thaliana*, *E. grandis* and *P. tomentosa*, the number of R2R3-MYB TFs in *P. massoniana* is significantly lower, and most of these genes perform unknown functions. This may be because *P. massoniana* studies rely on transcriptomic data [29,30,31,32]. R2R3-MYB play important roles in plant secondary metabolism, hormone response and growth and development [4,33]. *AtMYB7* is a key regulatory gene for the third layer of the SCW regulatory network in *A. thaliana*. Different plants express different types of *MYB7* TFs. In *P. glauca* and *P. taeda, PgMYB7* and *PtMYB7* belong to the 22nd subgroup defined based on *A. thaliana* sequences and are closely related to *AtMYB44* [34]. Cotton *GhMYB7* is grouped with *AtMYB26* and *AtMYB103* and could be involved in the regulation of SCW biosynthesis. The constitutive expression of *GhMYB7* in *A. thaliana* activated the expression of a suite of secondary cell wall biosynthesis-related genes (*CesA4*, *CesA7* and *CesA8*; *4CL1*, *CCoAOMT1* and *PAL1*), leading to the ectopic deposition of cellulose and lignin. [10]. *AtMYB7* is classified into subgroup 4 and acts as a repressor of lignin biosynthesis in *A. thaliana.* AtMYB7 repressed the expression of *4CL1*, while *AtMYB46* activated the expression of *4CL1* [16].

Studies have shown that the function and structure of MYB TFs are closely related [35]. In this study, the full-length *PmMYB7* cDNA was successfully isolated from *P. massoniana* for the first time. PmMYB7 protein showed high homology with the MYB7 proteins of Pinaceae. Sequence analysis showed that there are complete R2 and R3 repeat regions in the PmMYB7 protein sequence, so it belongs to the R2R3-MYB subfamily, and the primary structures of the R2 and R3 repeat regions (-W-(X19)-W-(X19)-W- and -F/I/L/M-(X18)-W-(X18)-W-) are consistent with those reported by Dubos [8]. In the R2 and R3 repeat regions, tryptophan folds the MYB domain into a helix-helix-turn-helix structure, which is consistent with the results of previous studies [36].

### 3.2. Analysis of PmMYB7 Expression Pattern

The expression of the *MYB7* genes differs significantly among different plants and tissues. The PmMYB7 protein of *P. massoniana* is closely evolutionarily related to the TcMYB64 protein of *T. chinensis*. Studies on *TcMYB64* expression in different tissues of *T. chinensis* have shown that the highest expression occurs in the phloem. In this study, the *PmMYB7* gene was expressed at a high level in old leaves, xylem, old stems and roots. The results of this study are consistent with those of the above studies [37]. These studies indicate that *PmMYB7* may be mainly expressed in phloem and lignified tissues and may be involved in SCW growth and development.

Plants quickly accumulate MeJA, ABA and SA upon injury, pathogenic attack and insect attack; these substances induce changes in the expression of genes related to stress resistance and promote the synthesis of resistance-related substances [38]. In this study, the expression of the *PmMYB7* gene peaked at different time points when *P. massoniana* was subjected to different abiotic stresses. Under ABA, ETH and GA treatment, the expression level of the *PmMYB7* gene peaked at 12 h; under H_2_O_2_, MeJA and PEG6000 treatment conditions, the expression level of *PmMYB7* peaked at 24 h. In *Salvia miltiorrhiza,* after a certain period of high-salt (250 mmol/L), MeJA (500 μM), SA (500 μM) and ABA (0.1 mmol/L) treatments, the expression of the *SmMYB7* gene was upregulated at different time points [39]; this time expression characteristic is similar to that of the *PmMYB7* gene in this study. However, *SbMYB7* expression in the root hair of *Scutellaria bornmuelleri* was induced by MeJA (100 μM) treatment for 24 h, but the modification of the level of expression was less than the *PmMYB7* gene under the MeJA stress. This result is different from the results of this study, probably because the concentration of MeJA used for induction (100 μM) was relatively low, and MYB7 exerts different degrees of regulation effects on different species [40]. Under 1 mM SA and wounding conditions, *PmMYB7* gene expression transiently increased again in *P. massoniana* seedlings, peaking at 12 h. *PmMYB7* expression was upregulated rapidly in a short duration, suggesting that *PmMYB7* may be involved in responses to SA and wounding stresses. Therefore, it can be speculated that *PmMYB7* participates in the synthesis of resistance-related substances, allowing *P. massoniana* to resist damage. This is consistent with the results that have been reported and shown, where the *SmMYB7* gene is involved in plant stress [39].

### 3.3. Protein–Protein Interaction Analysis

To understand the overall functioning of the cell, it is crucial to delineate the pairwise interactions between DNA and proteins, RNA and proteins or proteins and proteins. The discovery of protein interactions, including stable physical interactions (direct), functionally related protein interactions, transient binding interactions and information-based interactions (indirect), will help to explore protein function. There are diverse validation methods known for examining these interactions such as Y2H [41], Co-IP [42], etc., which provide information about the domains essential for sustaining the interaction or the proximity of the interactions [43].

In this study, The PSORT prediction results indicated that PmCCoAOMT2 protein is a mainly cytoplasmic protein. This result is consistent with the report that LrCCoAOMT was largely directed to the cytoplasm in *Lilium regale* [44]. However, the PmMYB7 protein is located in the nucleus. We speculate that when the PmMYB7 protein interacts with the PmCCoAOMT2 protein, the interaction site is located in the cytoplasm and nucleus. One explanation is that the function or localization of TFs may be affected by other TFs [45]. A more likely explanation is that PmMYB7 changes the subcellular localization of PmCCoAOMT2. PmCCoAOMT2 shows a change in its subcellular localization when interacting with proteins. For example, in *A. thaliana*, GL1 is located in the nucleus. However, in the co-localization study, the interaction between AtMYC and GL1 leads to GL1 localizing to the cytoplasm [46]. In *Pyrus bretschneideri*, the PbMC1a/1b protein was located in the cytoplasm and nucleus, and PbRD21 was only found in the nucleus. Through subcellular localization analysis, both PbMC1a/1b and PbRD21 were found in the nucleus, which may indicate the possibility of their interaction [47]. In *Heterodera schachtii*, Hs4E02 is located in the nucleus, causing RD21A to accumulate in different cell compartments, and interaction is observed in the cytoplasm as well as the nucleus [48]. Hence, when both proteins are present, a re-localization might occur.

Protein–protein interactions participate in various cellular processes at different temporal and spatial levels. Therefore, it is of great importance to study protein–protein interactions to understand molecular regulatory networks. We used PmMYB7 as a bait carrier to screen 47 proteins from the *P. massoniana* yeast library and predicted which of the thirty might interact with proteins involved in defense, stress response, protein biosynthesis, cell development, signal transduction and activation. The interaction between the PmCCoAMT2 and PmMYB7 proteins was verified by yeast two-hybrid and Co-IP techniques. CCoAOMT, a key enzyme involved in lignin synthesis, acts as a methyltransferase, catalyzing the conversion of Caffeoyl CoA into Feruloyl CoA (Figure 1). In *P. teada*, *CCoAOMT* plays a key role in G-type lignin synthesis [49]. Suppression of *PrCCoAOMT* expression in *P. radiata* tracheary element cultures resulted in reduced lignin contents of up to 20%, culminating in a lignin polymer containing p-hydroxyphenyl, catechyl and guaiacyl units [50]. In *P. massoniana*, *PmMYB4* was shown to bind to AC-box motifs and might directly activate the promoters of *PmCCoAOMT* genes involved in SCW biosynthesis [24]. In *A. thaliana*, AtMYB61 can activate the expression of CCoAOMT and affect xylem formation and xylem cell structure [51]. At present, there are no relevant reports on the function of *CCoAOMT2* in *P. massoniana*. Taken together, this study indicates that PmMYB7 can interact with PmCCoAOMT2 in *P. massoniana SCW*. Whether PmMYB7 or PmMYB7-PmCCoAOMT2 interactions regulate target gene transcription in the nucleus remains to be further studied. This work may help to better understand the possible functions of PmMYB7 and to provide data reference for further research.

Studies have found that two *xylosyltransferase* genes are involved in xyloglucan biosynthesis in *A. thaliana*. Disrupting both of these genes resulted in plants with a severe root hair phenotype that lacks detectable xyloglucan. Xyloglucans are the main polysaccharides in the primary cell walls of higher plants [52]. In *A. thaliana, XXT5* is a xyloglucan α-1,6-xylosyltransferase and functions in the biosynthesis of xyloglucan. xxt5 mutant seedlings demonstrated decreased xyloglucan quantity and reduced glucan backbone substitution with xylosyl residues [53]. In this study, the PmMYB7 protein did not interact with the PmXYXT1 protein. It is preliminarily hypothesized that *PmMYB7* does not participate in the synthesis of xyloglucan in the primary cell wall, but the precise biological activity of PmXYXT1 needs to be further studied.

## 4. Materials and Methods

### 4.1. Plant Materials

The experimental materials were 15-year-old trees of *P. massoniana* from Nanjing Forestry University and 2-year-old potted seedlings raised from the seed orchard of the Baisha state-owned forest farm, Fujian Province, China. *A. thaliana* (Col-0) and *N. benthamiana* were potted in the laboratory greenhouse at 24 °C under long-day conditions (16 h light: 8 h dark).

### 4.2. Full-Length Cloning of the cDNA Encoded by the PmMYB7 Gene

Total RNA was extracted from the organs/tissues of 15-year-old *P. massoniana*. The quality of the total RNA was tested by a Thermo NanoDrop 2000c (*Thermo* Scientific, Waltham, MA, USA) and agarose gel electrophoresis. First-strand cDNA was reverse transcribed from the extracted total RNA using EasyScript^®^ One-step gDNA Removal and cDNA Synthesis SuperMix (TransGen, Beijing, China). RACE cDNA was synthesized according to the SMARTer^®^ RACE cDNA Amplification Kit instruction (Takara, Dalian, China). Based on *MYB7* sequences from other species in the NCBI database, an expressed sequence tag (EST) of *MYB7* was retrieved from the existing *P. massoniana* transcriptome data. Specific primers were designed by using Primer Premier 5 software (Supplementary S4). The full-length complementary DNA (cDNA) of *MYB7* was cloned from *P. massoniana* using polymerase chain reaction (PCR) and rapid amplification of cDNA ends (RACE) technology (GenBank: MW579321). The PCR product was ligated into pEASY-Blunt clone vectors by the pEASY-Blunt Zero Cloning Kit (Transgen). The vectors were transformed into *Escherichia coli* cells (Weidi, Shanghai, China). The positive *E. coli* cells were selected and sequenced by Jie Li Biology (Shanghai, China).

### 4.3. Bioinformatics Analysis of the PmMYB7 Gene Coding Sequence

ExPASy (http://web.expasy.org/protparam/, accessed on 30 November 2021) online software was used to analyze the physicochemical properties of the PmMYB7 protein. The secondary structure of the PmMYB7 protein was predicted with the JPred4 (http://www.compbio.dundee.ac.uk/jpred4, accessed on 30 November 2021) [54]. The conserved domain of the PmMYB7 protein was analyzed in the NCBI Conserved Domain database (https://www.ncbi.nlm.nih.gov/Structure/cdd/wrpsb.cgi, accessed on 30 November 2021). The amino acid sequence encoded by the *PmMYB7* gene was compared with highly homologous sequences in the NCBI database, and the phylogenetic tree and alignment of the gene-encoded proteins were constructed using the MEGA X and DNAMAN software [55]. 

### 4.4. Quantitative Real-Time PCR (RT–qPCR) Analysis of PmMYB7 Gene Expression

Expression profiles of the *PmMYB7* gene were analyzed by using RT–qPCR. Different organs/tissues, including the roots (R), young stems (YS), old stems (OS), young leaves (YL), old leaves (OL), flowers (F) and xylem (X) and phloem (P) tissues of 15-year-old trees of *P. massoniana* and 2-year-old robust seedlings subjected to different treatments were collected for RNA extraction and reverse-transcription. The phloem and xylem were collected as described above [56]. The different abiotic stress treatments included 400 μM abscisic acid (ABA), 50 μM ethephon (ETH), 2 mM gibberellin (GA), 10 mM H_2_O_2_, 10 mM methyl jasmonate (MeJA), 15% polyethylene glycol (PEG 6000), 1 mM salicylic acid (SA) and wounding. The osmotic stress was induced by soaking the plants in PEG 6000, and the remaining abiotic stress and hormone treatments were applied by spraying the plant surfaces. The wounding treatment method was performed by cutting the upper half of the pine needles. Needles were collected at 0 h, 3 h, 6 h, 12 h and 24 h. To ensure the accuracy of the results, we performed three biological repetitions and three technical replicates. According to the obtained *PmMYB7* gene sequence, we designed qPCR primers (Supplementary S4), and used alpha-tubulin (*TUA*) (GenBank: KM496535.1) as the reference housekeeping gene. In different P. massoniana abiotic stresses, the most suitable reference gene for RT–qPCR analysis across all samples was TUA [57]. The reaction program was designed for PCR according to the instructions of the Hieff Unicon Universal Blue qPCR SYBGreen Master Mix Kit (Vazyme, Nanjing, China). The relative expression levels were calculated according to the 2^−ΔΔCt^ method [58].

### 4.5. Subcellular Localization of the PmMYB7 Protein 

To further study the *PmMYB7* gene, we used the bioinformatics analysis tool Cell-PLoc 2.0 (http://www.csbio.sjtu.edu.cn/bioinf/Cell-PLoc-2/, accessed on 30 November 2021) to predict the subcellular localization [59]. To verify the prediction, we used the pJIT166 vector to construct a recombinant plasmid containing a green fluorescent protein (GFP) marker and the PmMYB7 protein. The pJIT166 vector was digested at the HindIII and XbaI sites using QuickCut enzymes (Takara). Then, the *PmMYB7* coding sequence (without stop codon) was inserted into the pJIT166 vector. The 2x35S::PmMYB7-GFP fusion vector was transformed into the *E. coli* strain TOP10 (Weidi). Positive *E. coli* cells were cultured, and high-quality plasmids were extracted. The plasmids were transformed into leaf protoplasts of *A. thaliana* by the PEG-mediated method [60]. After 14 h, we observed and obtained images of the GFP fluorescence signal by using a laser scanning confocal microscope (LSM710, Zeiss, Jena, Germany).

### 4.6. Construction of the P. massoniana cDNA Yeast Library 

During plant growth and development, protein interactions are closely related to various biosynthesis processes in cells. To further explore the function of the *PmMYB7* gene, we constructed a cDNA yeast library of *P. massoniana* and screened the proteins that interacted with the PmMYB7 protein.

#### 4.6.1. Extraction of Total RNA and Isolation of mRNA

Fresh leaves, roots, flowers, shoots, stems and bark of *P. massoniana* were collected and ground into powder after freezing with liquid nitrogen. Total RNA was extracted according to the TRIzol extraction protocol (Takara). The quality of the RNAs was investigated by agarose gel electrophoresis and Thermo NanoDrop 2000c analysis. All the extracted RNA samples were mixed together for subsequent experiments. The mRNA samples were isolated by Oligo(dT) chromatography according to the instructions for the Oligotex mRNA Kit (Qiagen, Valencia, CA, USA). Then, 1 μL of the solution was used for electrophoresis.

#### 4.6.2. Synthesis of Double-Strand cDNAs and Addition of a 5′ Adapter

First-strand cDNA and second-strand cDNA were synthesized using the SuperScript Double-Stranded cDNA Synthesis Kit (Invitrogen, Shanghai, China). A 5′ adapter was added according to the protocol. Following detection via agarose gel electrophoresis, fragments larger than 1 k bp were excised from the gel and recovered, and the products were dissolved in 14 μL of DEPC ddH_2_O. The size of the double-stranded cDNA fragments was between 150 and 2000 bp. The ds cDNA was subsequently purified using a CHROMA SPIN TE-400 column instruction (Takara). The cDNA fragments ranged in size from 300 to 2000 bp (Supplementary S2), and the quality was up to standard. The ds cDNA was normalized using the Trimmer-Direct cDNA normalization kit protocol (Evrogen, Moscow, Russia). The 4 μL 4 × hybridization buffer was added into the ds cDNA and incubated at 98 °C for 2 min and then at 68 °C for 5 h. Then, 4 μL of 4 × DSN (duplex-specific nuclease) buffer and 0.2 μL DSN (1 U/μL) were added into the PCR tube and incubated at 68 °C for 3 min. The normalized product was extracted with phenol and chloroform once and finally diluted in 20 μL DEPC ddH2O.

#### 4.6.3. Ligation of the Double-Stranded cDNAs with Library Vectors

The cDNAs of *P. massoniana* were ligated to the pGADT7 vector to construct recombinant vectors. pGADT7 was digested at the NdeI and XhoI sites using QuickCut enzymes (Takara). The double-stranded cDNAs from the previous step were ligated with the linearized pGADT7 by homologous recombination. The ligation system contained 7 µL of cDNA, 3 µL of enzymatically linearized library vector DNA, 5 µL of All-Direct recombination enzyme (Biogene, Shanghai, China) and 5 µL of ddH_2_O. These components were incubated at 50 °C for 1 h. The recombination reaction was inactivated by adding 2 µL of proteinase K (Sigma–Aldrich, St. Louis, MO, USA), followed by the addition of 1 µL of 20 µg/µL glycogen (Sigma–Aldrich), 50 µL of 7.5 M NH_4_Ac and 375 µL of anhydrous ethanol. The components were mixed, and the reaction mixture was stored at −80 °C for at least 1 h. The recombination product was collected and suspended in 10 µL of DEPC ddH_2_O on ice. 

#### 4.6.4. Electroporation of the Recombinant Library into *Escherichia coli* TOP10 and *Saccharomyces cerevisiae* AH109

The recombinant vectors were electroporated into competent *E. coli* TOP10 cells. One-millimeter electroporation cuvettes were precooled at −80 °C for 20 min, after which 2.5 µL of the recombination product and 50 µL of competent cells of *E. coli* TOP10 were mixed in the cuvettes and incubated on ice for 45 min. Then, 1 mL of LB medium was quickly added to the cuvette. Liquid LB medium was added to the transformed bacteria to obtain a final volume of 5 mL in a new 15 mL centrifuge tube, and the tube was incubated at 37 °C for 1 h. The cultured bacteria (10 µL) were diluted 100-, 1000- and 10,000-fold and spread on LB agar plates with ampicillin (TransGen). The rest of the bacterial cultures were supplemented with glycerin at a final concentration of 20% and stored at −80 °C. The library plasmid was extracted at a high concentration using a plasmid extraction kit (Qiagen, Valencia, CA, USA) and used to transform *S. cerevisiae* AH109 (Weidi) via a similar method, replacing LB with YPDA [41].

#### 4.6.5. Identification of Library Capacity and the Average Length of the Inserted Fragments

Ten microliters of 10,000-fold diluted bacteria were used for LB plate counting the next day. The library capacity was calculated according to the formula CFU/mL = number of clones on the plate/10 µL × 1000 times × 1 × 10^3^ µL, and the total CFU of the library = CFU/mL × total volume of library (5 mL). A total of 22 single colonies from the plate were randomly selected for PCR amplification to determine the length of the inserted fragments of the library via agarose gel electrophoresis. Primers were designed for the pGADT7-Rec vector (Supplementary S4). The pGADT7-T vector was used as the positive control, while ddH_2_O was used as the negative control. The 22 positive colonies were sequenced at Jieli Biology (Shanghai, China). The results were blasted against the NCBI database to identify the characteristics and related functions in other species. The same calculation method described above was used to determine the yeast library capacity.

### 4.7. Analysis of the Autoactivation and Toxicity of Bait Vectors

To detect autoactivation of the *PmMYB7* gene, we used the pGBKT7 vector to construct a recombinant plasmid as a bait vector. The pGBKT7 vector was digested at the EcoRI and XbaI sites using QuickCut enzymes (Takara). The *PmMYB7* coding sequence was inserted into the pGBKT7 vector. Approximately 100 ng of pGBKT7-PmMYB7 bait vector and 100 ng of pGADT7 prey vector plasmids were cotransformed into *S. cerevisiae* AH109 cells to assess autoactivation and toxicity. pGBKT7-p53 and pGADT7-largeT were cotransformed as positive controls, and pGBKT7-laminC and pGADT7-largeT were cotransformed as negative controls. The transformed competent cells were cultured on SD/-Leu/-Trp (DDO) and SD/-Trp/-Leu/-His/-Ade (QDO) plates at 30 °C for 3–5 days. Three positive clones were randomly selected for HIS3 and ADE2 detection. The diameters and colors of the colonies were observed and recorded.

### 4.8. Screening and Identification of Positive Interactors and Retrieval of Interacting Proteins

The fresh colony of bait vector pGBKT7-PmMYB7 was selected and cultured in 50 mL SD/-Trp media at 30 °C, 250 rpm until the OD_600_ reached 0.8. Then, the AH109 transformed yeast cells were collected and resuspended in 5 mL SD/-Trp media, which was prepared for the mating with 1 mL cDNA library in 45 mL 2 × YPDA media at 30 °C, 50 rpm for 20 h. The growth of the zygotes was observed and recorded with an inverted microscope (XDS-1A, Shanghai, China). Based on the growth conditions of the positive and negative controls, yeast zygotes were collected and cultured on SD/-Leu/-Trp/-His (TDO) and SD/-Leu/-Trp/-His/X-α-Gal plates (TDO/X) at 30 °C for 4 days. The same positive and negative controls described above were used. The positive clone plasmids were extracted and transformed into *E. coli* TOP10 for identification by PCR and sequencing. The results were analyzed by BLAST against the GenBank database. Protein–protein interactions were predicted and network modeling was constructed by using the STRING 11.5 online tool (https://string-db.org/cgi/input?sessionId=bj86SJVlJU4A&input_page_show_search=on, accessed on 30 November 2021) and Cytoscape 3.9.0 software [61]. *A. thaliana* was used as a template. Based on the results of the screening library, we predicted the interactions of these proteins with the PmMYB7 protein. 

### 4.9. Point-to-Point Verification

The expression of genes is inseparable from the function of genes. On the basis of *PmMYB7* gene expression patterns and the yeast library screening results, we selected the caffeoyl CoA 3-O-methyltransferase-2 (CCoAOMT2) (GenBank: APX43199.1) protein and the β -1, 2-xylotransferase (XYXT1) (GenBank: XP_034920216.1) proteins to verify whether they interacted with PmMYB7 using a yeast two-hybrid assay. The *S. cerevisiae* expression vector pGADT7 was digested with the EcoRI and BamHI QuickCut enzymes (Takara). The ORFs of the *PmCCoAOMT2* and *PmXYXT1* genes were inserted into the digested vector by using recombinant technology. The transformation and culture methods used were similar to those described above. All of the reagents used in yeast cultivation were purchased from Clontech (Fitchburg, CA, USA) and Takara. The PCR products were sequenced at Ruiyuan Biotechnology (Nanjing, China).

### 4.10. Verify Protein Interactions Using Co-IP Assay

The pCAMBIA-N×myc-C×FLAG and pBinGFP2 (Beyotime, Shanghai, China) expression vectors were digested with restriction enzymes (*EcoRI* and *BamHI*, *KpnI* and *BamHI*). The full-length *PmMYB7* and *PmCCoAOMT2* ORFs were ligated into the linearized pCAMBIA-N×myc-C×FLAG and pBinGFP2 vectors according to the homologous recombination method of the In-Fusion HD Cloning Kit (Takara). The expected molecular masses of the recombinant PmMYB7-FLAG and GFP-PmCCoAOMT2 vectors were approximately 40.8 kD and 41.7 kD, respectively, and the molecular mass of GFP was 28 kD. We injected transformed GV3101 into the lower epidermis of *N. benthamiana* leaves for transient expression. Subsequently, total proteins were extracted from the leaves by a cell lysis solution (Qiagen) after 48 h. Pretreated GFP-tagged magnetic beads (Beyotime) were added to the transiently expressed protein solutions, and a GFP-tag was used for protein affinity purification. The purified proteins were added to a protein loading buffer and boiled. Finally, the proteins were separated by 4 to 20% SDA-PAGE, transferred to PVDF membranes, and subjected to Western blotting. Mouse anti-GFP and anti-FLAG (ABclonal, Wuhan, China) monoclonal antibodies were used as primary antibodies at 1:5000 dilutions, and the secondary antibody (ABclonal) was a goat anti-mouse HRP monoclonal antibody at 1:10,000 dilution. The protein bands were detected using ECL reagents and photographed. In addition, we have predicted PmCCoAOMT2 protein localization using the online software PSORT: Protein Subcellular Localization Prediction Tool (https://www.genscript.com/psort.html, accessed on 30 November 2021).

## 5. Conclusions

In this study, we cloned the full-length *PmMYB7* cDNA from *P. massoniana*. We found that the transcription factor *PmMYB7*, which belongs to the R2R3-MYB subfamily, is involved in the lignin biosynthesis in *P. massoniana*. *PmMYB7* was highly expressed in lignified tissues and under abiotic stress. The PmMYB7 protein caused no toxicity and was not autoactivated in the nucleus. Yeast two-hybrid and Co-IP assays demonstrated that the PmMYB7 protein could interact with the PmCCoAOMT2 protein but not with the PmXYXT1 protein, leading to the speculation that the PmMYB7 protein and the PmCCoAOMT2 protein play regulatory roles together in the cytoplasm and nucleus. In addition, we found that the *PmMYB7* gene may be involved in resistance, cell development, protein synthesis and signal transduction. However, these results require further experimental confirmation. Although protein–protein interaction data were obtained from yeast strains and tobacco, these data laid the foundation for further research on the *PmMYB7* gene and lignin synthesis in *P. massoniana*. We hope to verify some of the results in *P. massoniana*, which will be technically challenging in future studies. 

## Figures and Tables

**Table 1:** The functions of 47 proteins were predicted using the NCBI and STRING databases.

Protein ID	Species	Protein Name	Predicted Function
			**Resistance related**
XP_034896381.1	*P. alba*	Hyoscyamine 6-dioxygenase-like	Converts salicylic acid (SA) to 2,3-dihydroxybenzoic acid (2,3-DHBA); regulates negative defense-associated gene expressions.
XP_034894966.1	*P. alba*	Glucan endo-1,3-beta-glucosidase, basic isoform-like	May play a role in plant defense against pathogens.
XP_034927539.1	*P. alba*	Germin-like protein subfamily 1 member 13	May play a role in plant defense.
			**Abiotic stress related**
XP_034894692.1	*P. alba*	Osmotin-like protein OSM34	Involved in the ABA signaling pathway.
AAT12488.1	*P. alba x P. glandulosa*	Copper chaperone	Plays an important role in copper homeostasisby conferring tolerance to excessive copper levels and subclinical copper deficiency during vegetative stage.
XP_034890852.1	*P. alba*	Enhanced ethylene response protein 5 isoform X2	Ectopic expression of seed storage proteins 1; involved in the regulation of ethylene response.
XP_034906946.1	*P. alba*	Dehydration-responsive element-binding protein 2D-like	Encodes a member of the DREB subfamily A-2 of ERF/AP2 transcription factor family; involved in response to drought.
XP_034907399.1	*P. alba*	Chaperone protein dnaJ A6, chloroplastic-like	Cooperates with the chaperone HSC70 to assist protein folding and prevent protein aggregation in the chloroplast during heat stress.
XP_034931160.1	*P. alba*	40S ribosomal protein S12-like	Structural constituent of ribosome; involved in response to cadmium ions, response to salt stress and translation.
XP_006371633.2	*P. trichocarpa*	Uncharacterized protein	Wound-response family protein, including the DUF3774 domain.
XP_034917128.1	*P. alba*	BURP domain protein RD22-like	RD22 can alleviate salinity and osmotic stress.
ABK96099.1	*P. trichocarpa*	Clone WS0127_L05 unknown mRNA	Encodes a ferritin protein that is targeted to the chloroplast; gene expression is induced in response to iron overload and by nitric oxide.
			**Cell wall-related**
XP_034890693.1	*P. alba*	3-ketoacyl-CoA synthase 10	Contributes to cuticular wax and suberin biosynthesis.
APX43199.1	*P. tomentosa*	Caffeoyl CoA 3-O-methyltransferase 2	Methylates caffeoyl-CoA to feruloyl-CoA and 5-hydroxyferuloyl-CoA to sinapoyl-CoA; plays a role in the synthesis of feruloylated polysaccharides; involved in the reinforcement of the plant cell wall.
XP_034920216.1	*P. alba*	Beta-1,2-xylosyltransferase XYXT1	Glycosyltransferase family 61 protein; transferase activity; and transfers glycosyl groups.
XP_034927053.1	*P. alba*	Proline-rich protein-like	May have a specific role in modifying the cell-wall structure, specifically during seed germination, thus facilitating radicle protrusion.
			**Cell development and cellular component-related**
XP_034912044.1	*P. alba*	N-terminal acetyltransferase A complex catalytic subunit NAA10-like	Required for male gametocyte development, embryogenesis, suspensor development and the formation of the quiescent center (QC) in the root meristem.
XP_034916055.1	*P. alba*	40S ribosomal protein S5	Delay/disrupt cell-division processes and development at an early embryonic stage in the homozygous mutant.
XP_002313280.1	*P. trichocarpa*	60S ribosomal protein L6	Structural constituent of ribosome; involved in translation.
XP_034915118.1	*P. alba*	40S ribosomal protein S15-like	Structural constituent of ribosome; involved in translation.
XP_024440441.1	*Populus trichocarpa*	40S ribosomal protein S30	Structural constituent of ribosome; involved in translation.
XP_034922981.1	*P. alba*	60S ribosomal protein L13a-4	Structural constituent of ribosome; involved in translation.
XP_034918559.1	*P. alba*	60S ribosomal protein L36-2-like	Structural constituent of ribosome; involved in translation.
			**Catalytic activity and protein synthesis-related**
XP_024456673.1	*P. trichocarpa*	Probable protein Phosphatase 2C 9	Serine/threonine phosphatase activity, catalytic activity.
XP_034909248.1	*P. alba*	Adenylate kinase isoenzyme 6 homolog isoform X1	Catalyze the reversible transfer of the terminal phosphate group between nucleoside triphosphates and monophosphates.
XP_034914042.1	*P. alba*	Ubiquitin-conjugating enzyme E2 28	Accept the ubiquitin from the E1 complex and catalyze its covalentattachment to other proteins.
XP_034932113.1	*P. alba*	ATP-citrate synthase beta chain protein 1	ATP citrate-lyase that is used for the elongation of fatty acids and biosynthesis of isoprenoids, flavonoids and malonated derivatives.
XP_034903658.1	*P. alba*	Nuclear pore complex protein NUP98A isoform X2	NUP96 and NUP98 are not translated as polyproteins.
XP_034914799.1	*P. alba*	Fructose-bisphosphate aldolase 6, cytosolic-like	Fructose-bisphosphate aldolase that plays a key role in glycolysis and gluconeogenesis.
AXY97901.1	*P. tomentosa*	Enolase 2	In particular, enolase 2 (ENO2) is a glycolytic enzyme that is present almost exclusively in neurons and neuroendocrine cells.
XP_034932781.1	*P. alba*	Polyubiquitin-like	Polyubiquitin and ubiquitin-like signals share common recognition sites on proteasomal subunit Rpn1
XP_034899578.1	*P. alba*	Probable carboxylesterase 2	Carboxylesterase acting on esters with varying acyl chain length.
XP_034889715.1	*P. alba*	Rhodanese-like domain-containing protein 17 isoform X2	Cysteine persulfide intermediate.
XP_034889386.1	*P. alba*	Wound-responsive protein GWIN3-like	Kunitz trypsin inhibitor TI3; most inhibit serine proteases (families S1 and S8).
XP_034916340.1	*P. alba*	Transmembrane 9 superfamily member 8-like	Endomembrane protein 70 protein family; located integral to membrane, Golgi apparatus, plasma membrane, plant-type cell wall.
XP_034899278.1	*P. alba*	Nuclear/nucleolar GTPase 2	GTPase involved in pre-60S ribosomal subunit maturation.
XP_034896238.1	*P. alba*	Vacuolar protein sorting-associated protein 2 homolog 1-like	Component of the ESCRT-III complex, which is required for multivesicular bodies (MVBs) formation.
XP_034892684.1	*P. alba*	Uncharacterized protein isoform X1	DNA-directed RNA polymerase subunit beta–beta protein.
XP_006343015.2	*Solanum tuberosum*	RWD domain-containing protein 1isoform X1	RWD domain-containing protein1 is also known as RWDD or RWDD1 and is sometimes seen expressed as DFRP2.
ABK92558.1	*P. trichocarpa*	Clone PX0015_H16 unknown mRNA	Glycine hydroxymethyltransferase; encodes a serine hydroxymethyltransferase maximally expressed in root.
			**Unknown protein**
XP_034922861.1	*P. alba*	Vegetative cell wall protein gp1-like isoform X2	Located in endomembrane system.
XP_002301538.3	*P. trichocarpa*	Protein rolling stone isoform X2	Located in endomembrane system.
XP_034894915.1	*P. alba*	Uncharacterized protein	Unknown protein.
XP_034891584.1	*P. alba*	Uncharacterized protein	Unknown protein.
XP_034913569.1	*P. alba*	Uncharacterized protein isoform X1	Uncharacterized protein.
ABK92753.1	*P. trichocarpa*	Clone PX0019_O01 unknown	Uncharacterized protein.
ABK96721.1	*P. trichocarpa x P. deltoides*	Clone WS0137_I01 unknown	Uncharacterized protein.

Note: Bold black fonts represent functional categories.

## Data Availability

Not applicable.

## Supplementary Materials

The following supporting information can be downloaded at: https://www.mdpi.com/article/10.3390/ijms23042079/s1.

## References

[1] Zhang J., Nieminen K., Serra J.A., Helariutta Y. (2014). The formation of wood and its control. Curr. Opin. Plant Biol..

[2] Sundell D., Street N.R., Kumar M., Mellerowicz E.J., Kucukoglu M., Johnsson C., Kumar V., Mannapperuma C., Delhomme N., Nilsson O. (2017). Aspwood: High-spatial-resolution transcriptome profiles reveal uncharacterized modularity of wood formation in *Populus tremula*. Plant Cell.

[3] Houston K., Tucker M.R., Chowdhury J., Shirley N., Little A. (2016). The plant cell wall: A complex and dynamic structure as revealed by the responses of genes under stress conditions. Front. Plant Sci..

[4] Zhang J., Xie M., Tuskan G.A., Muchero W., Chen J.G. (2018). Recent advances in the transcriptional regulation of secondary cell wall biosynthesis in the woody plants. Front. Plant Sci..

[5] Liu C.J., Miao Y.C., Zhang K.W. (2011). Sequestration and transport of lignin monomeric precursors. Molecules.

[6] Liu Q., Luo L., Zheng L. (2018). Lignins: Biosynthesis and biological functions in plants. Int. J. Mol. Sci..

[7] Wang Q., Dai X., Pang H., Cheng Y., Huang X., Li H., Yan X., Lu F., Wei H., Sederoff R.R. (2021). BEL1-like homeodomain protein BLH6a is a negative regulator of *CAld5H2* in sinapyl alcohol monolignol biosynthesis in poplar. Front. Plant Sci..

[8] Dubos C., Stracke R., Grotewold E., Weisshaar B., Martin C., Lepiniec L. (2010). MYB transcription factors in *Arabidopsis*. Trends Plant Sci..

[9] Goicoechea M., Lacombe E., Legay S., Mihaljevic S., Rech P., Jauneau A., Lapierre C., Pollet B., Verhaegen D., Chaubet-Gigot N. (2005). *EgMYB2*, a new transcriptional activator from *Eucalyptus* xylem, regulates secondary cell wall formation and lignin biosynthesis. Plant J..

[10] Huang J., Chen F., Wu S., Li J., Xu W. (2016). Cotton *GhMYB7* is predominantly expressed in developing fibers and regulates secondary cell wall biosynthesis in transgenic *Arabidopsis*. Sci. China Life Sci..

[11] Kim W.C., Ko J.H., Kim J.Y., Kim J., Bae H.J., Han K.H. (2013). *MYB46* directly regulates the gene expression of secondary wall-associated cellulose synthases in *Arabidopsis*. Plant J..

[12] Li C., Wang X., Lu W., Liu R., Tian Q., Sun Y., Luo K. (2014). A poplar R2R3-MYB transcription factor, *PtrMYB152*, is involved in regulation of lignin biosynthesis during secondary cell wall formation. Plant Cell Tissue Organ Cult..

[13] Zhao Q., Richard A.D. (2011). Transcriptional networks for lignin biosynthesis: More complex than we thought?. Trends Plant Sci..

[14] Kim W.C., Kim J.Y., Ko J.H., Kang H., Han K.H. (2014). Identification of direct targets of transcription factor *MYB46* provides insights into the transcriptional regulation of secondary wall biosynthesis. Plant Mol. Biol..

[15] Karpinska B., Karlsson M., Srivastava M., Stenberg A., Schrader J., Sterky F., Bhalerao R., Wingsle G. (2004). MYB transcription factors are differentially expressed and regulated during secondary vascular tissue development in hybrid aspen. Plant Mol. Biol..

[16] Kim W.C. (2016). AtMYB7 acts as a repressor of lignin biosynthesis in *Arabidopsis*. J. Appl. Biol. Chem..

[17] Legay S., Sivadon P., Blervacq A.S., Pavy N., Baghdady A., Tremblay L., Levasseur C., Ladouce N., Lapierre C., Séguin A. (2010). *EgMYB1*, an R2R3 MYB transcription factor from eucalyptus negatively regulates secondary cell wall formation in *Arabidopsis* and poplar. New Phytol..

[18] Patzlaff A., Newman L.J., Dubos C., Whetten R.W., Smith C., McInnis S., Bevan M.W., Sederoff R.R., Campbell M.M. (2003). Characterisation of *PtMYB1*, an R2R3-MYB from pine xylem. Plant Mol. Biol..

[19] Bedon F., Grima-Pettenati J., Mackay J. (2007). Conifer R2R3-MYB transcription factors: Sequence analyses and gene expression in wood-forming tissues of white spruce (*Picea glauca*). BMC Plant Biol..

[20] Bomal C., Bedon F., Caron S., Mansfield S.D., Levasseur C., Cooke J.E.K., Blais S., Tremblay L., Morency M.-J., Pavy N. (2008). Involvement of *Pinus taeda MYB1* and *MYB8* in phenylpropanoid metabolism and secondary cell wall biogenesis: A comparative in planta analysis. J. Exp. Bot..

[21] Patzlaff A., McInnis S., Courtenay A., Surman C., Newman L.J., Smith C., Bevan M.W., Mansfield S., Whetten R.W., Sederoff R.R. (2003). Characterisation of a pine MYB that regulates lignification. Plant J..

[22] Pascual M.B., Llebrés M.T., Craven-Bartle B., Cañas R.A., Cánovas F.M., Ávila C. (2018). *PpNAC1*, a main regulator of phenylalanine biosynthesis and utilization in maritime pine. Plant Biotechnol. J..

[23] Li R., Chen P., Zhu L., Wu F., Chen Y., Zhu P., Ji K. (2021). Characterization and function of the 1-deoxy-D-xylose-5-phosphate synthase (*DXS*) gene related to terpenoid synthesis in *Pinus massoniana*. Int. J. Mol. Sci..

[24] Yao S., Chen P., Yu Y., Zhang M., Wang D., Liu J., Hao Q., Ji K. (2021). *PmMYB4*, a Transcriptional activator from *Pinus massoniana*, regulates secondary cell wall formation and lignin biosynthesis. Forests.

[25] Studer M.H., Demartini J.D., Davis M.F., Sykes R.W., Davison B., Keller M., Tuskan G., Wyman C.E. (2011). Lignin content in natural *Populus* variants affects sugar release. Proc. Natl. Acad. Sci. USA.

[26] Li Q., Song J., Peng S., Wang J.P., Qu G.Z., Sederoff R.R., Chiang V.L. (2014). Plant biotechnology for lignocellulosic biofuel production. Plant Biotechnol. J..

[27] Chen P., Wu X., Wei Q., Wu X., Ji K. (2017). Research progress of lignin synthesis gene in Pinaceae. J. Nanjing For. Univ. (Nat. Sci. Ed.).

[28] Wu F., Sun X., Zou B., Zhu P., Lin N., Lin J., Ji K. (2019). Transcriptional analysis of Masson Pine (*Pinus massoniana*) under high CO_2_ stress. Genes.

[29] Fan F., Wang Q., Wen X., Ding G. (2020). Transcriptome-wide identification and expression profiling of *Pinus massoniana* MYB transcription factors responding to phosphorus deficiency. J. For. Res..

[30] Matus J.T., Aquea F., Arce-Johnson P. (2008). Analysis of the grape MYB R2R3 subfamily reveals expanded wine quality-related clades and conserved gene structure organization across *Vitis* and *Arabidopsis* genomes. BMC Plant Biol..

[31] Wilkins O., Nahal H., Foong J., Provart N.J., Campbell M.M. (2009). Expansion and diversification of the *Populus* R2R3-MYB family of transcription factors. Plant Physiol..

[32] Soler M., Camargo E.L.O., Carocha V., Cassan-Wang H., San Clemente H., Savelli B., Hefer C., Paiva J., Myburg A.A., Grima-Pettenati J. (2015). The *Eucalyptus grandis* R2R3-MYB transcription factor family: Evidence for woody growth-related evolution and function. New Phytol..

[33] Lea U.S., Slimestad R., Smedvig P., Lillo C. (2007). Nitrogen deficiency enhances expression of specific MYB and bHLH transcription factors and accumulation of end products in the flavonoid pathway. Planta.

[34] Kranz H.D., Denekamp M., Greco R., Jin H., Leyva A., Meissner R.C., Petroni K., Urzainqui A., Bevan M., Martin C. (1998). Towards functional characterisation of the members of the R2R3-MYB gene family from *Arabidopsis thaliana*. Plant J..

[35] Liu J., Osbourn A., Ma P. (2015). MYB transcription factors as regulators of phenylpropanoid metabolism in plants. Mol. Plant.

[36] Frampton J. (2004). Myb Transcription Factors: Their Role in Growth, Differentiation and Disease.

[37] Hu X., Zhang L., Wilson I., Shao F., Qiu D. (2020). The R2R3-MYB transcription factor family in *Taxus chinensis*: Identification, characterization, expression profiling and posttranscriptional regulation analysis. PeerJ.

[38] Dong X. (1998). SA, JA, ethylene, and disease resistance in plants. Curr. Opin. Plant Biol..

[39] Zhu X., Cui C., Zhou H., Wu Y., Wang Z. (2015). Cloning and expression analysis of the transcription factor *SmMYB7* from *Salvia miltiorrhiza* Bunge. Genom. Appl. Biol..

[40] Gharari Z., Bagheri K., Danafar H., Sharafi A. (2020). Enhanced flavonoid production in hairy root cultures of *Scutellaria bornmuelleri* by elicitor induced over-expression of *MYB7* and *FNSП2* genes. Plant Physiol. Biochem..

[41] Uetz P., Giot L., Cagney G., Mansfield T.A., Judson R.S., Knight J.R., Lockshon D., Narayan V., Srinivasan M., Pochart P. (2000). A comprehensive analysis of protein-protein interactions in *Saccharomyces cerevisiae*. Nature.

[42] Takahashi Y. (2015). Co-immunoprecipitation from transfected cells. Protein-Protein Interactions.

[43] Li L.G., Osakabe Y.K. (1999). Secondary xylem-specific expressin of caffeoyl-coenzyme A 3-O-methyltransferase plays an important role in the methylation pathway associated with lignin biosynthetic in loblloly pine. Plant Mol. Biol..

[44] Fu Y., Zhu Y., Yang W., Xu W., Li Q., Chen M., Yang L. (2020). Isolation and functional identification of a *Botrytis cinerea*-responsive caffeoyl-CoA O-methyltransferase gene from *Lilium regale* wilson. Plant Physiol. Biochem..

[45] Pesch M., Schultheiß I., Digiuni S., Uhrig J.F., Hülskamp M. (2013). Mutual control of intracellular localisation of the patterning proteins *AtMYC1*, *GL1* and *TRY/CPC* in *Arabidopsis*. Development.

[46] Yu N., Cai W., Wang S., Shan C., Wang L., Chen X. (2010). Temporal control of trichome distribution by microRNA156-targeted SPL genes in *Arabidopsis thaliana*. Plant Cell.

[47] Gong X., Xie Z., Qi K., Zhao L., Yuan Y., Xu J., Rui W., Shiratake K., Bao J., Khanizadeh S. (2020). *PbMC1a/1b* regulates lignification during stone cell development in pear (*Pyrus bretschneideri*) fruit. Hort. Res..

[48] Pogorelko G.V., Juvale P.S., Rutter W.B., Hütten M., Maier T.R., Hewezi T., Paulus J., van der Hoorn R.A., Grundler F.M., Siddique S. (2019). Re-targeting of a plant defense protease by a cyst nematode effector. Plant J..

[49] Chopra K., Burdak B., Sharma K., Kembhavi A., Mande S.C., Chauhan R. (2020). CoRNeA: A pipeline to decrypt the inter-protein interfaces from amino acid sequence information. Biomolecules.

[50] Wagner A., Tobimatsu Y., Phillips L., Flint H., Torr K., Donaldson L., Pears L., Ralph J. (2011). CCoAOMT suppression modifies lignin composition in *Pinus radiata*. Plant J..

[51] Romano J.M., Dubos C., Prouse M.B., Wilkins O., Hong H., Poole M., Kang K., Li E., Douglas C.J., Western T.L. (2012). *AtMYB61*, an R2R3-MYB transcription factor, functions as a pleiotropic regulator via a small gene network. New Phytol..

[52] Cavalier D.M., Lerouxel O., Neumetzler L., Yamauchi K., Reinecke A., Freshour G., Zabotina O.A., Hahn M.G., Burgert I., Pauly M. (2008). Disrupting two *Arabidopsis thaliana* xylosyltransferase genes results in plants deficient in xyloglucan, a major primary cell wall component. Plant Cell.

[53] Zabotina O.A., Van De Wen W.T., Freshour G., Drakakaki G., Cavalier D., Mouille G., Hahn M.G., Keegstra K., Raikhel N.V. (2008). *Arabidopsis* XXT5 gene encodes a putative α-1,6-xylosyltransferase that is involved in xyloglucan biosynthesis. Plant J..

[54] Drozdetskiy A., Cole C., Procter J., Barton G.J. (2015). JPred4: A protein secondary structure prediction server. Nucleic Acids Res..

[55] Kumar S., Stecher G., Li M., Knyaz C., Tamura K. (2018). MEGA X: Molecular evolutionary genetics analysis across computing platforms. Mol. Biol. Evol..

[56] Li Q., Min D., Wang J.P.Y., Peszlen I., Horvath L., Nishimura Y., Jameel H., Chang H.-M., Chiang V.L. (2011). Down-regulation of glycosyltransferase 8D genes in *Populus trichocarpa* caused reduced mechanical strength and xylan content in wood. Tree Physiol..

[57] Zhu P., Ma Y., Zhu L., Chen Y., Li R., Kongshu J., Ji K. (2019). Selection of suitable reference genes in *Pinus massoniana* lamb. under different abiotic stresses for qPCR normalization. Forests.

[58] Livak K.J., Schmittgen T.D. (2001). Analysis of relative gene expression data using real-time quantitative PCR and the 2 ^−ΔΔCt^ method. Methods.

[59] Chou K., Shen H. (2010). Cell-PLoc: An improved package of Web-servers for predicting subcellular localization of proteins in various organisms. Nat. Protoc..

[60] Yoo S.D., Cho Y.H., Sheen J. (2007). *Arabidopsis* mesophyll protoplasts: A versatile cell system for transient gene expression analysis. Nat. Protoc..

[61] Szklarczyk D., Franceschini A., Wyder S., Forslund K., Heller D., Huerta-Cepas J., Simonovic M., Roth A., Santos A., Tsafou K.P. (2015). STRING v10: Protein-protein interaction networks, integrated over the tree of life. Nucleic Acids Res..

