# Supplementary material for: Characterization and Interaction Analysis of the Secondary Cell Wall Synthesis-Related Transcription Factor PmMYB7 in Pinus massoniana Lamb
Source: Int J Mol Sci. 2022 Feb 14;23(4):2079. doi: 10.3390/ijms23042079 (PMC8877852; doi:10.3390/ijms23042079)
Supplement: Supplementary file 1 [file ijms-23-02079-s001.zip › Supplementary 1.pdf]

Supplementary 1

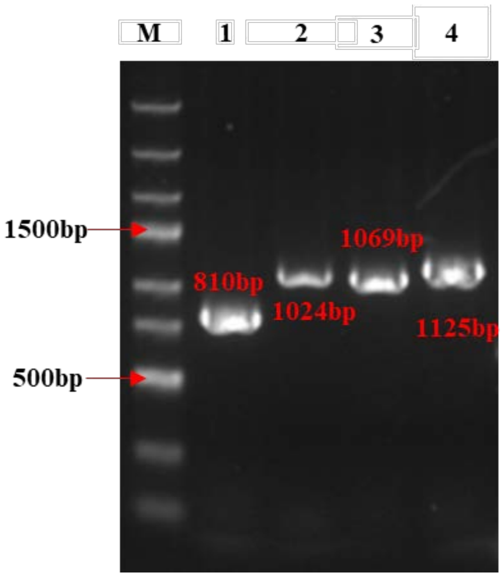

Figure 1 Cloning of *PmMYB7*gene

Note: (M: NormalRun^{TM} 250 bp-II DNA ladder). 1. PCR amplification product of the intermediate fragment; 2. PCR amplification product of the 3' RACE fragment; 3. PCR amplification product of the 5' RACE fragment. 4. Cloning of the ORF fragment.

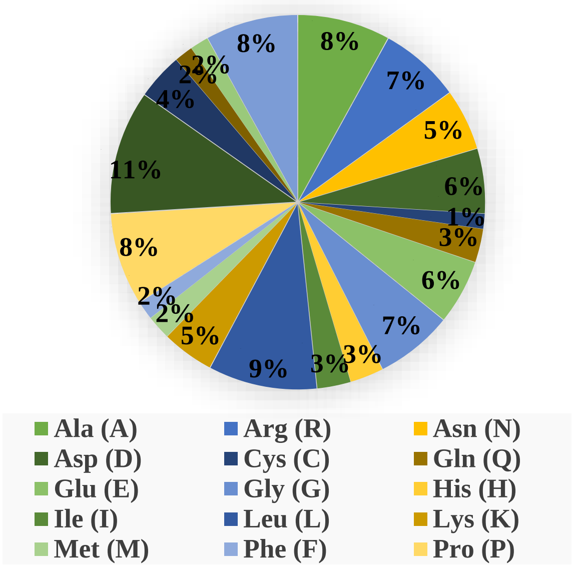

Figure 2 Proportions of amino acids in the PmMYB7 protein.

The predicted secondary structure includes chiefly 60.43% random coil and 30.21% α-helices. There are 42 negatively charged residues (Asp + Glu) and 43 positively charged residues (Arg + Lys) in the protein structure. The 20 amino acids and their proportions are shown in Fig 2.
