# Supplementary material for: Characterization and Interaction Analysis of the Secondary Cell Wall Synthesis-Related Transcription Factor PmMYB7 in Pinus massoniana Lamb
Source: Int J Mol Sci. 2022 Feb 14;23(4):2079. doi: 10.3390/ijms23042079 (PMC8877852; doi:10.3390/ijms23042079)
Supplement: Supplementary file 1 [file ijms-23-02079-s001.zip › Supplementary 2.pdf]

# Supplementary 2

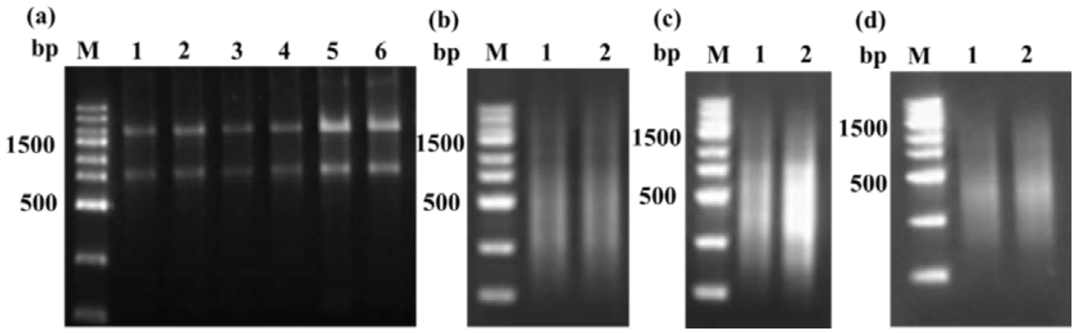


**Figure 1. (a)** Total RNA extracted from *P. massoniana*. M: DL 5000 DNA Marker; RNA samples from 1: leaves, 2: roots, 3: flowers, 4: shoots, 5: stems, 6: bark. **(b, c d)** M: NormalRunTM 250 bp-II DNA ladder; 1: mRNA or cDNA from *P. massoniana*; 2: Positive control.

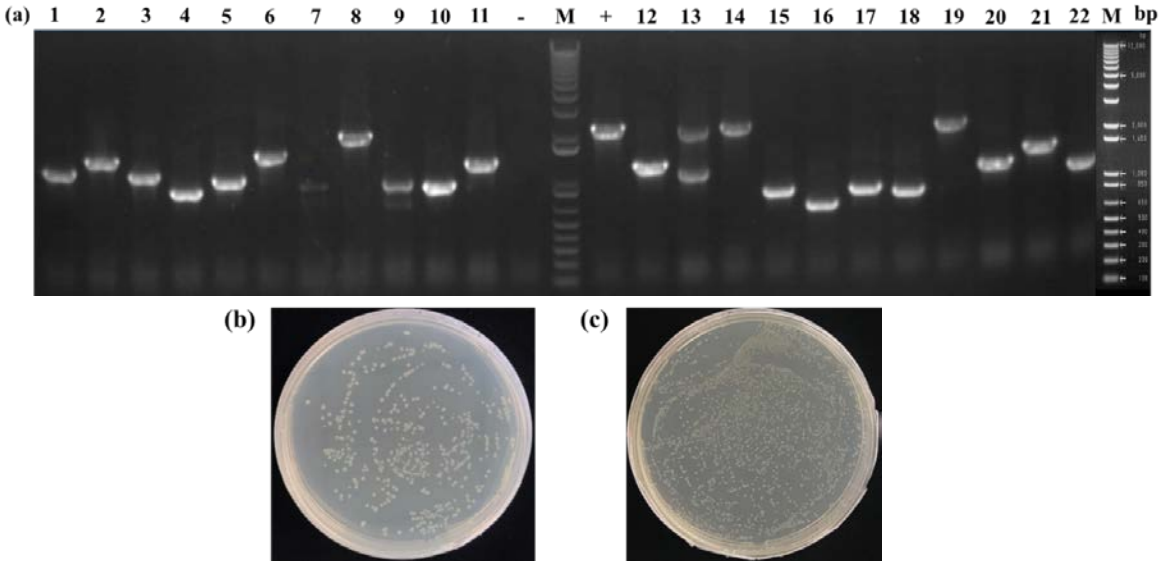


**Figure 2.** Quantification of the library by sequencing positive colonies and plate counting. **(a)** Agarose gel electrophoresis of PCR products from 22 randomly selected colonies. M: DL 12000 DNA Marker; 1–22: PCR products of 22 colonies; +: pGADT7-T vector as positive control; −: ddH2O as negative control. **(b)** Plate counting of 10000-fold diluted bacteria from the E. coli library. **(c)** Plate counting of 1000-fold diluted yeast cells from the yeast library.
