# Supplementary material for: Characterization and Interaction Analysis of the Secondary Cell Wall Synthesis-Related Transcription Factor PmMYB7 in Pinus massoniana Lamb
Source: Int J Mol Sci. 2022 Feb 14;23(4):2079. doi: 10.3390/ijms23042079 (PMC8877852; doi:10.3390/ijms23042079)
Supplement: Supplementary file 1 [file ijms-23-02079-s001.zip › Supplementary 3.pdf]

>1-4

AATCACTACTGTGATGATAACGAAGACAATTCCAAGGAGCTGAAAATCATCATCATCATCATCATCAT
CAATCAGCTAAGATTAAGAATTATGATAAACCTAAACCGTTCGGTTTCGATTTGGATCGTATTATCAT
TATTTCTCTCACTCTTCCTTTCTCAAGCTGAAGTTATAACCCTAACTCCTGAAACATTCTCTGACAAGG
TGAAGGAGAAAGACACGGCATGGTTTGTCAAATTTTGTGTGCCATGGTGTAAGCATTGTAAGAATTTG
GGGACCTTGTGGGAAGAGGTTGGGAAGGCAATGGAGGGGGAAGATGAAATAGAGGTTGGAGAAGTT
GACTGCGGCGCCAGCAAATCTGTCTGTTCTAAAGCTGATATCCACTCTTACCCTACTTTTAAGCTCTTT
TTTGATGGAGAAGAAGTTGCTAAATATCAAGGGCCAAGAGATGTTGAATCTCTTAAAGCATTCGTTTT
AGATGAAGCAGAAAAGGCAGCAGCTAAAGCACAGCTTGGCTATGATAAAGATTTGTGAGCGTTTGTA
AGGcAAATCTCCTTTTCaATATTTCCATCTGTTTGTCATAGTGATTTATCCAGAGACGTTGTGGACATCC
CCATTGGGGCAGTGGAGCCTCACAATGGAATAAATTTGATTTTGCAAACGCCCCAGTTCAGATTACCT
TGTGCGTTGTAGCAGATAATATTTGACAATATGCCCATTTTGGTTGTCATCTTGTAGGAGGAGGTCTG
CAACAATGGATGGAAACCCCAGTCAGGAATATAACAAAAACTTGTAGGCACTGATAATGGATCCATT
GCGAAGTTATATCATCCTTACACAGGAATTTCAACATAATGTCTTGAACAGTTTAATTATATTAGCGA
GTGTAATTGGTAACATATTTTCTCTTCTAAACAAGCGAAGTTAGTGAAAAAAAAAAAAAAAAAAAAA
AA

PREDICTED: Populus alba protein disulfide-isomerase 5-1 (LOC118034619), transcript variant X1, mRNA. XM_035039958.1

Descriptions | Graphic Summary | Alignments | Taxonomy

Sequences producing significant alignments Download New Manage Columns Show 100

select all *0 sequences selected* GenBank Graphics Distance tree of results New MSA Viewer

Description	Scientific Name	Common Name	Taxid	Max Score	Total Score	Query Cover	E value	Per. Ident	Acc. Len	Accession
PREDICTED: Populus alba protein disulfide-isomerase 5-1 (LOC118034619), transcript va...	Populus...	white po...	43335	1711	1711	97%	0.0	99.89%	992	XM_035039958.1
PREDICTED: Populus trichocarpa protein disulfide-isomerase 5-1 (LOC7491175), transcri...	Populus...	black co...	3694	1437	1437	94%	0.0	95.70%	978	XM_002313510.3
PREDICTED: Populus euphratica protein disulfide-isomerase 5-1 (LOC105114437), transc...	Populus...	Euphrat...	75702	1399	1399	97%	0.0	94.04%	995	XM_011010982.1
PREDICTED: Populus alba protein disulfide-isomerase 5-1 (LOC118034619), transcript va...	Populus...	white po...	43335	1000	1363	77%	0.0	99.82%	801	XM_035039959.1
PREDICTED: Populus euphratica protein disulfide-isomerase 5-1 (LOC105114437), transc...	Populus...	Euphrat...	75702	850	1346	94%	0.0	95.40%	957	XM_011010983.1
PREDICTED: Populus trichocarpa protein disulfide-isomerase 5-1 (LOC7491175), transcri...	Populus...	black co...	3694	699	699	45%	0.0	95.89%	867	XM_024608065.1
PREDICTED: Populus euphratica protein disulfide-isomerase 5-1 (LOC105114437), transc...	Populus...	Euphrat...	75702	662	662	45%	0.0	94.52%	827	XM_011010981.1
PREDICTED: Jatropha curcas protein disulfide-isomerase 5-1 (LOC105628125), mRNA	Jatroph...	NA	180498	431	431	39%	7e-116	87.43%	1000	XM_012209472.3

>2-1

GGAGACAATCACTCCGACCTGACTATATCAAGATGTTTGTATTGGATGAAGCAGATGAAATGCTCTCA
AGAGGATTCAAGGATCAGATATATGATATTTTCCAATTACTGCCCCCAAAGATTCAGGTTGGGGTCTT
CTCTGCTACTATGCCACCTGAGGCCCTAGAAATTACGAGGAAGTTCATGAATAAACCTGTGAGGATTT
TAGTGAAGCGTGATGAGCTTACCCTTGAGGGTATCAAGCAATTCTATGTTAATGTTGACAAGGAGGA
ATGGAAGCTTGAGACACTTTGTGATCTATATGAGACCTTGGCAATAACCCAAAGTGTTATCTTTGTGA
ACACCAGGCGCAAGGTGGATTGGCTCACAGACAAGATGCGCAGTCGTGATCACACAGTCTCTGCCAC
TCATGGAGATATGGACCAAAACACAAGGGACATTATTATGCGAGAATTCCGCTCTGGTTCCTCTCGTG
TGCTGATCACTACTGATCTGTTGGCCCGTGGTATTGATGTCCAGCAAGTCTCACTTGTGATCAATTATG
ATCTCCCTACCCAGCCAGAGAACTACCTCCATCGTATTGGTCGTAGTGGACGTTTTGGAAGGAAGGGT
GTTGCTATAAACTTTGTTACCAGGGACGATGAAAGAATGCTTTCTGACATCCAGAGATTCTATAATGT

AACGGTTGAGGAGCTGCCATCAAATGTTGCTGATCTTCTTTGAAGCAATTTTGGTTCTCCAAGGTAAA
TGGCTGAGCTTGGAAGTTAAAACTATTATGCTGTTTGCAGTTTTGGGGCCTCTATTTAGCATGGATTG
GACTAGCCCTGCAGGTTTGGTTCTGAGCATTTGAGATTGGGGTCAATCCCATTTCGGGTGCAGGAGTC
CCTTGTGTTAAATTTTGTTGCAAGTTTGTTTTTGCTTTTTCCTTTTTGTGTCGGAGTAAAATTTGCAGAA
TTATGGTAGTGTCCATCATAATATTTTACTCTCTGCGTGGTTTTTCATTTTATGCAAGGGAATTTCATG
TAGGTAAAAAACATATGATAAAGTCATTATGTTATAATATTGGGCTCTCCGTTGCTGTTCTCTTGAGC
AATAAAAAAAAAAAAAAAAAAAAAAAAA

PREDICTED: Populus alba eukaryotic initiation factor 4A-9-like (LOC118030765), transcript variant X2, mRNA.
XM_035035016.1

Descriptions | Graphic Summary | Alignments | Taxonomy

Sequences producing significant alignments — Download — New Select columns — Show 100

select all *100 sequences selected* — GenBank — Graphics — Distance tree of results — New MSA Viewer

Description	Scientific Name	Max Score	Total Score	Query Cover	E value	Per. Ident	Acc. Len	Accession
PREDICTED: Populus alba eukaryotic initiation factor 4A-9-like (LOC118030765), transcript variant X2, ...	Populus alba	2004	2004	97%	0.0	99.73%	1823	XM_035035016.1
PREDICTED: Populus alba eukaryotic initiation factor 4A-9-like (LOC118030765), transcript variant X1, ...	Populus alba	2004	2004	97%	0.0	99.73%	1832	XM_035035015.1
Populus trichocarpa clone WS0111_L18 unknown mRNA	Populus trichoc...	1969	1969	99%	0.0	98.83%	1779	EF145043.1
PREDICTED: Populus trichocarpa eukaryotic initiation factor 4A-9 (LOC7470684), transcript variant X3, ...	Populus trichoc...	1956	1956	97%	0.0	98.99%	1822	XM_024604079.1
PREDICTED: Populus trichocarpa eukaryotic initiation factor 4A-9 (LOC7470684), transcript variant X2, ...	Populus trichoc...	1956	1956	97%	0.0	98.99%	1822	XM_024604078.1
PREDICTED: Populus trichocarpa eukaryotic initiation factor 4A-9 (LOC7470684), transcript variant X1, ...	Populus trichoc...	1956	1956	97%	0.0	98.99%	1823	XM_002308509.3
PREDICTED: Populus euphratica eukaryotic initiation factor 4A-9-like (LOC105122170), transcript varia...	Populus euphra...	1912	1912	97%	0.0	98.35%	1812	XM_011021112.1
PREDICTED: Populus euphratica eukaryotic initiation factor 4A-9-like (LOC105122170), transcript varia...	Populus euphra...	1912	1912	97%	0.0	98.35%	1814	XM_011021111.1
PREDICTED: Populus trichocarpa eukaryotic initiation factor 4A-15 (LOC112325060), transcript variant ...	Populus trichoc...	1428	1428	96%	0.0	90.53%	1808	XM_024590479.1

>2-3
AGAGCTACATAGACAAGCAGATGCAATCTTCCACCTCTATGGAACGGTCACTGCTCTTCTCTCTTTTCT
TGCTCTTCTCAATATCCATCGTTGCCCAATCTACTGTCCCTTCAAATTCCACATTTAAGAAAGTCAATA
CTGGAGAGTGGGCGGAAGCCATTTCAGAGTACCGTTCAGATTTTCGTGCCCTGGACATCTCTACCTCC
GTTTTCCAAGTTTGCTTTTATAACACCACCCCTAATGCATTCACTCTCGCAATACGTATGGGTACGAGG
AGGTCACCGGCAGTGAGGCGGTTTGTTTGGGAAGCCAACCGAGGCAACCCAGTTGGTGAAGATGCCA
CTCTCACTTTTGGTGAGGACGGAAACCTTATCTTGGCCGATGCTGATGGCAGGGTTGCTTGGCAAACC
AACACTGCCGACAAAGGTGTTGTAGGGTTGCAAATGCTACCGAATGGTAACATGGTGCTTCATGACT
CTAAGGGCAATTTCATCTGGCAAAGTTTTGATTATCCCACTGATACTCTTTTGGTGGGTCAATCTCTTC
GTGTTGGAGCCGTAACCAGGCTTGTGAGTCGAGCCTCTGACAAAATGAACACAAACGGAGCCTATAG
TCTGGTATTAGAACCCAAAAGAATAGCCATGTACTATAAGAGTCCAAACTCTCCCAAGCCATACGTCT
ACTACACATCTGATTTGTTTAGTATACAGAAAGGGCGTCTACAATATGTGAGGCTAATCAATTCGGCT
AATGACCTGAGTCTTCAGTTCTCTACCGGTGGTGGACCCTTGCTCTCTAAGCCCGATTTCAACAGCAC
GTTGTCATTTCTCCGGCTTGGGGTAGATGGCAATCTTAGAGTCTACAGTTTCAACAACCAGGAAACTT
CAGCTTCTTGGGACGTGACTTTCACTCTTTTCTCCAAAGATGTAGGTGTTTGGGAAAGTGAGTGTCAA
TTACCAGAGAAATGTGGCAAGTTTGGGCTCTGTGAGGACAGCCAATGCGTTGGTTGCCCGTTACCAA
ATGGGCTCGGGAACTGGACCGAGAGCTGTGAACCTGTGAAGGTGACTGTATGTAACAAGAACTTCCA
TTACTATAAACTAGAAGGGGTTGATCATTCCATGAGCAAGTATGGAAAAGGAAATGGACCCTTGAAG
GAGAACGATTGTGAGAAGGAATGTTCCAGTGACTGCAAGTGTTCAGGTTACTTTTACAACACAAAGA
CATCTATGTGTTGGATTGCTTATGATCTGCAAACTCTGACGAGGGTTGCAAATTCTACACATGTGGGT

TACATAAAAGTGCCGAATCACCAGTGAAGCTGGCCTGCTTGTTTTCACCAAGGCATGTAGTAGAATAC
TGAGATCGTTTACTTGGCGAGGCTAGTTTATCGCTTGCTGACATTTCTGGAGCAGTGTTTGCTTGCAAG
TGCCTGATCTCTCTGCTTACTGTTTCCTTCTGTAGCAAAATAAAAGTGGTAATAAAAAACATTTGAGC
ATTTCAAAAAAAAAAAAAAAAAAA

PREDICTED: Populus alba epidermis-specific secreted glycoprotein EP1-like (LOC118043395), mRNA. XM_035051360.1

Descriptions | Graphic Summary | Alignments | Taxonomy

Sequences producing significant alignments Download New Select columns Show 100

select all *27 sequences selected* GenBank Graphics Distance tree of results New MSA Viewer

	Description	Scientific Name	Max Score	Total Score	Query Cover	E value	Per. Ident	Acc. Len	Accession
☑	PREDICTED: Populus alba epidermis-specific secreted glycoprotein EP1-like (LOC118043395), mRNA	Populus alba	2760	2760	98%	0.0	99.87%	1542	XM_035051360.1
☑	PREDICTED: Populus trichocarpa epidermis-specific secreted glycoprotein EP1 (LOC18095492), mRNA	Populus trichoc...	2398	2398	98%	0.0	95.46%	1660	XM_006370091.2
☑	PREDICTED: Populus euphratica epidermis-specific secreted glycoprotein EP1-like (LOC105117520), ...	Populus euphra...	2337	2337	98%	0.0	94.57%	1561	XM_011015205.1
☑	PREDICTED: Populus euphratica epidermis-specific secreted glycoprotein EP1-like (LOC105111758), ...	Populus euphra...	2320	2320	98%	0.0	94.38%	1553	XM_011007210.1
☑	PREDICTED: Populus trichocarpa epidermis-specific secreted glycoprotein EP1 (LOC18103363), mRNA	Populus trichoc...	1458	1458	88%	0.0	86.30%	1545	XM_024581031.1
☑	Populus trichocarpa clone POP038-L21, complete sequence	Populus trichoc...	1458	2681	88%	0.0	86.30%	106705	AC214468.1
☑	PREDICTED: Populus euphratica epidermis-specific secreted glycoprotein EP1-like (LOC105129278), ...	Populus euphra...	1413	1413	88%	0.0	85.75%	1553	XM_011031270.1
☑	PREDICTED: Populus alba epidermis-specific secreted glycoprotein EP1-like (LOC118038309), mRNA	Populus alba	1408	1408	88%	0.0	85.67%	1560	XM_035044633.1
☑	PREDICTED: Populus trichocarpa epidermis-specific secreted glycoprotein EP1 (LOC7455947), mRNA	Populus trichoc...	636	636	82%	2e-177	76.20%	1532	XM_002316823.3
☑	PREDICTED: Populus alba epidermis-specific secreted glycoprotein EP1-like (LOC118038308), mRNA	Populus alba	621	621	81%	6e-173	76.03%	1645	XM_035044632.1
☑	PREDICTED: Populus trichocarpa epidermis-specific secreted glycoprotein EP1 (LOC7472351), mRNA	Populus trichoc...	586	586	81%	2e-162	75.55%	1413	XM_024580973.1
☑	PREDICTED: Populus alba epidermis-specific secreted glycoprotein EP1-like (LOC118038307), mRNA	Populus alba	564	564	81%	1e-155	75.22%	1478	XM_035044631.1
☑	PREDICTED: Populus euphratica epidermis-specific secreted glycoprotein EP1-like (LOC105129277), ...	Populus euphra...	536	536	71%	2e-147	75.92%	1503	XM_011031269.1

>2-5
ACTTTGAAATCCGAAACAATTGTCCTTACACTGTGTGGGCCGCAGCCTCACCTGGCGGTGGGCGCCGT
CTAGACCGTGGCCAAACTTGGAATCTTAACGTGCCTGCTGGCACGTCCATGGCTCGTATATGGGGCCG
GACAAATTGTAACTTTGATGGTAGCGGTCAGGGTCGTTGCCAAACCGGGGATTGCACCGGTGGCCTA
CAGTGCAAGGGCTGGGGTGTCCCTCCCAACACTCTAGCAGAATATGCGTTAAATCAGTTTGGTGACTT
GGATTTTTATGATATATCGCTTGTTGATGGATTTAATATCCCTATGGAATTTAGTCCAACATCAAGCGG
TGGCTCAGGGAAGTGTCAAGCGCTTCTGTGCACAGCAGATATTAATGGGCAATGTCCTAATGAATTG
AGGGCTCCTGGGGGGTGTAATAACCCATGCACTGTGTTCAAAACTAACGAATACTGCTGCACTAATG
GGCAGGGGAGCTGTGGCCCTACCTATTTCTCAAGGTTTTTAAGGATAGGTGCCCTACTTCTTACAGC
TATCCCCAGGATGACCCTTCGAGCACATTTACATGCCCTGGCGGGACCAACTATAGGGTTGTCTTTTG
CCCTCGGGGGTCTCCTCATTTCCCCTTGGAGATGGTTGAAGAACAGCGTGCAGAGTAAGCAAGAAGC
TAAGAACATAGCTGACGACTTGATTTATAATAAATATTATGTGCTGCCTTGAAAACTTGGTGAGTTGA
GGTCCGTGTGTTATGTACGTATGGCTGTGTGTATCTTTTTTCTTTTTCTTTTTCCTGCTAAAAGAACAAA
AAACGAGTCTGTGTTTTGTTCTTGCCTGTTTCCATGCTGGGATGTAATAAAAATTCCAGTGAAATAAA
GAAATGTTTAATCACAAAAAAAAAAAAAAAAAAAAAAAAAA

PREDICTED: Populus alba osmotin-like protein OSM34 (LOC118033711), mRNA. XM_035038801.1

Descriptions | Graphic Summary | Alignments | Taxonomy

Sequences producing significant alignments Download New Select columns Show 100

select all *26 sequences selected* GenBank Graphics Distance tree of results New MSA Viewer

	Description	Scientific Name	Max Score	Total Score	Query Cover	E value	Per. Ident	Acc. Len	Accession
☑	PREDICTED: Populus alba osmotin-like protein OSM34 (LOC118033711), mRNA	Populus alba	1489	1489	97%	0.0	96.39%	1108	XM_035038801.1
☑	PREDICTED: Populus trichocarpa osmotin-like protein OSM34 (LOC7472761), mRNA	Populus trichocarpa	1445	1445	97%	0.0	95.77%	1024	XM_002299485.3
☑	PREDICTED: Populus alba osmotin-like protein OSM34 (LOC118033665), mRNA	Populus alba	1151	1151	91%	0.0	91.51%	1024	XM_035038738.1
☑	PREDICTED: Populus euphratica osmotin-like protein OSM34 (LOC105129023), mRNA	Populus euphratica	1136	1136	96%	0.0	90.08%	1039	XM_011030919.1
☑	PREDICTED: Populus euphratica osmotin-like protein OSM34 (LOC105120343), parti...	Populus euphratica	1120	1311	90%	0.0	96.20%	899	XM_011018508.1
☑	PREDICTED: Populus euphratica osmotin-like protein OSM34 (LOC105129073), mRNA	Populus euphratica	1114	1305	90%	0.0	96.05%	973	XM_011031003.1
☑	Populus deltoides pathogenesis-related thaumatin (PR52-1) gene, partial cds	Populus deltoides	1083	1083	70%	0.0	96.61%	723	GU129189.1
☑	PREDICTED: Populus trichocarpa osmotin-like protein OSM34 (LOC7479328), mRNA	Populus trichocarpa	1053	1053	91%	0.0	89.70%	1017	XM_002299511.3
☑	Populus trichocarpa x Populus deltoides clone WS0132_O22 unknown mRNA	Populus trichocarpa x Populus de...	998	998	69%	0.0	94.90%	641	EF148520.1
☑	Populus balsamifera pathogenesis-related thaumatin (PR52-1) gene, partial cds	Populus balsamifera	627	627	41%	8e-175	95.87%	461	GU129190.1
☑	Populus nigra pathogenesis-related thaumatin (PR52-1) gene, partial cds	Populus nigra	616	616	41%	2e-171	94.57%	461	GU129188.1
☑	Hevea brasiliensis cultivar RRII-105 osmotin 2 gene, complete cds	Hevea brasiliensis	477	477	69%	9e-130	80.34%	813	MN683521.1

>3-1

AGGCAAGACCATTACACTTGAGGTGGAGAGTTCTGACACCATTGATAATGTGAAAGCTAAGATCCAA
GACAAGGAGGGTATCCCGCCAGACCAGCAGAGACTCATCTTTGCCGGCAAGCAGCTTGAGGATGGCC
GTACCCTTGCGGATTATAACATCCAGAAGGAGTCCACCCTCCATTTGGTCCTTCGTCTTCGTGGTGGT
ATGCAGATCTTTGTGAAGACCCTCACAGGAAAGACCATCACCTTGGAGGTGGAGAGCTCTGATACCA
TTGACAATGTGAAAGCCAAGATCCAAGACAAGGAAGGCATCCCCCCAGACCAGCAGAGGTTGATTTT
TGCTGGGAAGCAGCTTGAAGATGGGAGGACTCTTGCTGACTACAACATTCAGAAGGAGTCAACCCTT
CACCTGGTGCTGCGTCTCCGTGGAGGAATGCAGATTTTTGTCAAGACTTTGACCGGAAAGACCATCAC
CCTGGAGGTCGAGAGCTCGGACACCATTGACAACGTGAAGGCCAAGATCCAGGACAAGGAAGGTAT
TCCCCAGACCAACAAAGGTTGATCTTCGCTGGAAAGCAGTTGGAAGATGGGCGCACCTTGGCTGACT
ACAATATCCAGAAGGAATCCACACTTCACCTTGTCCTTCGTCTCCGTGGTGGTTTTTAAGATGTGCTGT
TCATGTTGTCCAAGATAATGTCTTGGTTTATATGCTATTACTCTGGATTATGGTTCTCTTGTGTGTTCA
GTGGCCATCTTTGAATGGTTGCTTTTAATAATCTGTTGAACTCTTTTTAAAAAAAAAAAAAAAAAAAA
AA

PREDICTED: Populus alba polyubiquitin-like (LOC118062990), mRNA. XM_035076890.1

Descriptions | Graphic Summary | Alignments | Taxonomy

Sequences producing significant alignments

Download | New Select columns | Show 100

select all 100 sequences selected

GenBank Graphics Distance tree of results New MSA Viewer

Description	Scientific Name	Max Score	Total Score	Query Cover	E value	Per. Ident	Acc. Len	Accession
PREDICTED: Populus alba polyubiquitin-like (LOC118062990), mRNA	Populus alba	1380	1380	97%	0.0	98.11%	1532	XM_035076890.1
Populus trichocarpa clone WS0125_N24 unknown mRNA	Populus trichocarpa	1341	1950	97%	0.0	97.23%	1438	EF147905.1
PREDICTED: Populus trichocarpa polyubiquitin (LOC18095912), mRNA	Populus trichocarpa	1336	1950	97%	0.0	97.10%	1544	XM_006370571.2
Populus trichocarpa ubiquitin mRNA, complete cds	Populus trichocarpa	1336	1944	97%	0.0	97.10%	1411	FJ438462.1
Populus trichocarpa clone WS0127_P02 unknown mRNA	Populus trichocarpa	1336	1950	97%	0.0	97.10%	1435	EF148144.1
Populus trichocarpa clone PX0011_N13 unknown mRNA	Populus trichocarpa	1336	1950	97%	0.0	97.10%	1448	EF144248.1
PREDICTED: Populus euphratica polyubiquitin (LOC105142396), mRNA	Populus euphratica	1301	1701	97%	0.0	96.34%	1484	XM_011049998.1
Populus EST from leave	Populus tremula x Popul...	1243	1243	97%	0.0	94.87%	1003	CU224824.1
Populus EST from leave	Populus tremula x Popul...	1131	1131	84%	0.0	96.37%	1246	CU222992.1
Poplar cDNA sequences	Populus trichocarpa x Po...	1118	1118	81%	0.0	97.27%	712	CT029634.1
Poplar cDNA sequences	Populus trichocarpa x Po...	1118	1118	81%	0.0	97.27%	712	CT029633.1
Poplar cDNA sequences	Populus trichocarpa x Po...	1118	1118	81%	0.0	97.27%	713	CT029613.1
Poplar cDNA sequences	Populus trichocarpa x Po...	1083	1083	77%	0.0	97.77%	677	CT029614.1

>4-2

ATTGGGATCAAAAGATCATCATCACAATCATCATTAATGTCATTATCACAATCATCATCACTTCCCAT
CAATCCATGCCCACCTTCCAAATTCCCTATTTTGGTTTCTTGATCATTTCCTGCTGTCTCAGTATGGTCA
ATGGGAGCTGAAAGTGGGGTTCTTGGAGACAAACTAGTAGCTGAAAGTGGAGAAGAACACAAGCCC
TTGTCTGCTGAAATATTGGGTTGCTCGGATTCTTTTTCTTCAGGCTTTTGAACTGCTTGTTGAACCTTGT
TTCTTGTACTACCGGCAAGTGAGTTCCGGTGAGTCGGACGTGGGTGTGTGTGATCGCCTGTGTAGCTA
ACAATAAACATGTTTGGATCTGTGTTGCTCCTCTCCACTTGCTTTCTTGCTGCACACCCTTTTGAGCTG
CTGCATCTGTAGTAGTTCCTGTACAAGAAAAAGAATCGAATTTTAGCCATGTTTAGTGAGAAGAATCA
AGATATTTTTAAAGAAAAGAAGGAAAAAAAA

PREDICTED: Populus alba probable WRKY transcription factor 27 (LOC118037092), mRNA. XM_035042975.1

Descriptions | Graphic Summary | Alignments | Taxonomy

Sequences producing significant alignments

Download | New Select columns | Show 100

select all 14 sequences selected

GenBank Graphics Distance tree of results New MSA Viewer

Description	Scientific Name	Max Score	Total Score	Query Cover	E value	Per. Ident	Acc. Len	Accession
PREDICTED: Populus alba probable WRKY transcription factor 27 (LOC118037092), mRNA	Populus alba	778	778	84%	0.0	99.30%	1740	XM_035042975.1
PREDICTED: Populus trichocarpa probable WRKY transcription factor 27 (LOC18106446), mR...	Populus trichocarpa	778	778	84%	0.0	99.30%	1724	XM_006372308.2
PREDICTED: Populus euphratica probable WRKY transcription factor 27 (LOC105140777), m...	Populus euphratica	736	736	84%	0.0	97.67%	2003	XM_011047753.1
PREDICTED: Populus trichocarpa probable WRKY transcription factor 27 (LOC7468350), mRNA	Populus trichocarpa	479	479	84%	1e-130	86.70%	1727	XM_002305235.3
PREDICTED: Populus alba probable WRKY transcription factor 27 (LOC118030321), mRNA	Populus alba	455	455	74%	2e-123	88.45%	1547	XM_035034378.1
PREDICTED: Populus euphratica uncharacterized LOC105120336 (LOC105120336), ncRNA	Populus euphratica	388	388	74%	2e-103	85.30%	1348	XR_841307.1
Malus domestica genome assembly, chromosome: 7	Malus domestica	132	132	33%	4e-26	80.70%	35442570	OU744997.1
Malus domestica genome assembly, chromosome: 7	Malus domestica	132	132	33%	4e-26	80.70%	35756407	OU744959.1
Malus domestica genome assembly, chromosome: 7	Malus domestica	132	132	33%	4e-26	80.70%	36203908	OU744548.1
Malus sylvestris genome assembly, chromosome: 7	Malus sylvestris	132	132	33%	4e-26	80.70%	35530347	OU696509.1
Malus domestica genome assembly, chromosome: 7	Malus domestica	132	132	33%	4e-26	80.70%	35293072	OU696683.1
PREDICTED: Malus domestica probable WRKY transcription factor 27 (LOC108173713), mRNA	Malus domestica	128	128	31%	5e-25	80.86%	1813	XM_017333280.2
PREDICTED: Tarenaya hassleriana probable WRKY transcription factor 27 (LOC104811943),...	Tarenaya hassleriana	126	126	28%	2e-24	82.19%	1495	XM_010538831.1

>4-5

AGTAAGGAACTGGAGAAGGAGCCCAAGTTCCTGAAGAACGGTGATGCTGGTATGATTAAGATGATTC
CCACCAAGCCCATGGTGGTGGAGACTTTCTCAGCGTATCCTCCACTCGGTCGTTTTGCTGTGAGGGAC
ATGCGCCAGACCGTTGCTGTGGGAGTTATCAAGAACGTGGAGAAGAAGGATCCCTCTGGTGCTAAGG
TGACCAAATCTGCTGCCAAGAAGGGTGCCAAGTGAACCGTGCAGGTTGATGCACTAAGAAGCTTTAA
TAAAAATCAAGAGTGGGTTCTTGTTACTGGTCCATTTCTTGGATGTCCATCTGTTCATTATGAGAACTG
TAGTCTTAAGTCATCACCTTCATTGCAAGGCTTTTGTTCACAGAACTGGGTTCTTGATAGGCGGTGGC
GGGGTTTTGGTCTTGATGTTTTTTCCGAGTCATTGTTGTGTCTCCCATGTTGAGAATTTTAATCTGTTTT
AATTTTCACTTGCTCTGCTGAGAGTTTAGTATTTTGCTGTGATGTTAAGTACCCATGAATACTGTTTAC
AATTTTACTTGTCTAGTTTTTGTTCGTCTACTTAGTTTCTACCTATGCCTTTGTAGAAGAGTGATATTCT
GAATGTTTTTTCAAAAAAAAAAAAAAAAAAAAAA

PREDICTED: Populus alba elongation factor 1-alpha (LOC118059947), mRNA. XM_035072992.1

Descriptions | Graphic Summary | Alignments | Taxonomy

Sequences producing significant alignments — Download — New Select columns — Show 100

select all *100 sequences selected* — GenBank — Graphics — Distance tree of results — New MSA Viewer

Description	Scientific Name	Max Score	Total Score	Query Cover	E value	Per. Ident	Acc. Len	Accession
PREDICTED: Populus alba elongation factor 1-alpha (LOC118059947), mRNA	Populus alba	1107	1107	96%	0.0	98.56%	1988	XM_035072992.1
PREDICTED: Populus trichocarpa elongation factor 1-alpha-like (LOC112328984), transcript variant X2,...	Populus trichoc...	1035	1035	96%	0.0	96.49%	1859	XM_024610823.1
PREDICTED: Populus trichocarpa elongation factor 1-alpha-like (LOC112328984), transcript variant X1,...	Populus trichoc...	1035	1035	96%	0.0	96.49%	1916	XM_024610822.1
PREDICTED: Populus trichocarpa elongation factor 1-alpha (LOC7489636), mRNA	Populus trichoc...	1029	1029	96%	0.0	96.33%	1915	XM_002316315.3
Populus trichocarpa clone JGIACSB1089-D10, complete sequence	Populus trichoc...	989	1827	96%	0.0	95.37%	39340	AC208942.1
PREDICTED: Populus euphratica elongation factor 1-alpha (LOC105137070), mRNA	Populus euphra...	915	915	90%	0.0	94.87%	1825	XM_011042649.1
Populus trichocarpa x Populus deltoides translation elongation factor 1 alpha mRNA, partial cds	Populus trichoc...	575	575	58%	2e-159	92.86%	481	GQ253565.1
PREDICTED: Populus euphratica elongation factor 1-alpha-like (LOC105130805), mRNA	Populus euphra...	521	521	71%	3e-143	86.97%	1404	XM_011033484.1
PREDICTED: Populus trichocarpa elongation factor 1-alpha (LOC7457880), mRNA	Populus trichoc...	516	516	71%	1e-141	86.75%	1825	XM_002311071.3
Populus trichocarpa clone WS01122_O05 unknown mRNA	Populus trichoc...	516	516	71%	1e-141	86.75%	1742	EF145289.1
PREDICTED: Populus alba elongation factor 1-alpha-like (LOC118052437), mRNA	Populus alba	499	499	72%	1e-136	85.77%	1805	XM_035063421.1
PREDICTED: Populus alba elongation factor 1-alpha (LOC118059944), mRNA	Populus alba	475	475	67%	2e-129	87.05%	1864	XM_035072988.1
PREDICTED: Populus euphratica elongation factor 1-alpha-like (LOC105137066), transcript variant X1,...	Populus euphra...	473	473	67%	8e-129	86.36%	1856	XM_011042642.1
