# Supplementary material for: Characterization and Interaction Analysis of the Secondary Cell Wall Synthesis-Related Transcription Factor PmMYB7 in Pinus massoniana Lamb
Source: Int J Mol Sci. 2022 Feb 14;23(4):2079. doi: 10.3390/ijms23042079 (PMC8877852; doi:10.3390/ijms23042079)
Supplement: Supplementary file 1 [file ijms-23-02079-s001.zip › Supplementary 4.pdf]

Supplementary 4

Primer name	Primer sequence
MYB7-F：	5′- CAGGCTACGGTGGTGCAATCAG -3′
MYB7-R:	5′- CATCAGCGCCAGAAGGTCGCTG -3′
MYB7-5'GSP1:	5′- GGAGTCCACTTCCCAGCTGCTACAG -3′
MYB7-5'GSP2:	5′- CTCTCATGTATTCCACCACCGGCGGAAT -3′
MYB7-3'GSP1:	5′- CAGTCGGTGTCGATCCGCCGACCT -3′
MYB7-3'GSP2:	5′- CTCCCCGGCCGCACCGACAACGCGATCA -3′
MYB7-ORF-F:	5′- ATGGCGATGAGCAATGGGAGAT -3′
MYB7-ORF-R:	5′- TCATTTGTTGAGATGCAGGGCG -3′
MYB7-Q-F:	5′- CGGTGGTGGAATACATGAGA -3′
MYB7-Q-R:	5′- GCTGGTGATGACTGGACATA -3′
pJIT166-PmMYB7-F:	5′- tggagaggacagcccaagcttATGGCGATGAGCAATGGGA -3′
pJIT166-PmMYB7-R:	5′- gctcaccatggatcctctagaTTTGTTGAGATGCAGGGCG -3′
pGADT7-Rec-F:	5′- CACTAACAGTAGCAACGGTCCG -3′
pGADT7-Rec-R:	5′- GGCAAGGTAGACAAGCCGACAAC -3′
pGBKT7-PmMYB7-F:	5′- atggccatggaggccgaattcATGGCGATGAGCAATGGGA -3′
pGBKT7-PmMYB7-R:	5′- ccgctgcaggtcgacggatccTCATTTGTTGAGATGCAGGGC -3′
pGADT7-PmCCoAOMT2-F:	5′- gccatggaggccagtgaattcATGGACATCAACAGAGAGAACTATGAA -3′
pGADT7-PmCCoAOMT2-R:	5′- cagctcgagctcgatggatccTCACTTGATCCGACGGCAGA -3′
pGADT7-PmXYXT1-F:	5′- gccatggaggccagtgaattcATGGTGCAGTACTTTAGGTATCATCAG -3′
pGADT7-PmXYXT1-R:	5′- cagctcgagctcgatggatccGAGATGAGATCTGCGGTTCATTT -3′
pBinGFP2-F	5′- TGAGCGAAACCCTATAAGAACCCT -3′
pBinGFP2-R	5′- CAGGTAGTGGTTGTCGGGCAGCAG -3′
pCAMBIA-N×myc-C×FLAG-F	5′- ACTAATATCAGAGGAAGATTTGGA -3′
pCAMBIA-N×myc-C×FLAG-R	5′- AACATTTGGCAATAAAGTTTCTTA -3′
